# TP53 drives abscopal effect by secretion of senescence-associated molecular signals in non-small cell lung cancer

**DOI:** 10.1186/s13046-021-01883-0

**Published:** 2021-03-05

**Authors:** Anna Tesei, Chiara Arienti, Gianluca Bossi, Spartaco Santi, Ilaria De Santis, Alessandro Bevilacqua, Michele Zanoni, Sara Pignatta, Michela Cortesi, Alice Zamagni, Gianluca Storci, Massimiliano Bonafè, Anna Sarnelli, Antonino Romeo, Carola Cavallo, Armando Bartolazzi, Stefania Rossi, Antonella Soriani, Lidia Strigari

**Affiliations:** 1 Biosciences Laboratory, Istituto Scientifico Romagnolo per lo Studio e la Cura dei Tumori (IRST) IRCCS, Meldola, Italy; 2 Oncogenomic and Epigenetic Unit, IRCCS - Regina Elena National Cancer Institute, Rome, Italy; 3 CNR Institute of Molecular Genetics “Luigi Luca Cavalli-Sforza”, Bologna, Italy; 4 IRCCS Istituto Ortopedico Rizzoli, Bologna, Italy; 5 Department of Medical and Surgical Sciences (DIMEC), Alma Mater Studiorum, University of Bologna, I-40138 Bologna, Emilia Romagna Italy; 6 Interdepartmental Centre Alma Mater Research Institute on Global Challenges and Climate Change (Alma Climate), University of Bologna, I-40126 Bologna, Emilia Romagna Italy; 7 Department of Computer Science & Engineering (DISI), University of Bologna, Bologna, Italy; 8 Advanced Research Centre on Electronic Systems for Information & Communication Technologies ‘E. De Castro’ (ARCES), University of Bologna, Bologna, Italy; 9 Department of Experimental, Diagnostic and Specialty Medicine, University of Bologna, Bologna, Italy; 10 Medical Physics Unit, Istituto Scientifico Romagnolo per lo Studio e la Cura dei Tumori (IRST) IRCCS, Meldola, Italy; 11 Radiotherapy Unit, Istituto Scientifico Romagnolo per lo Studio e la Cura dei Tumori (IRST) IRCCS, Meldola, Italy; 12 Laboratorio RAMSES, Rizzoli Research, Innovation & Technology Department (RIT), IRCCS Istituto Ortopedico Rizzoli, Bologna, Italy; 13 Pathology Research laboratory, Sant’Andrea Hospital, Rome, Italy; 14 Department of Oncology and Molecular Medicine, Istituto Superiore di Sanità, Rome, Italy; 15 Laboratory of Medical Physics and Expert Systems, IRCCS - Regina Elena National Cancer Institute, Rome, Italy; 16 Department of Medical Physics, IRCCS University Hospital of Bologna, via Massarenti 9, 40138 Bologna, Italy

**Keywords:** Abscopal effect, Non-small cell lung cancer, TP53, Cellular senescence, Extracellular vesicles, DNA:RNA hybrids, Retrotransposon

## Abstract

**Background:**

Recent developments in abscopal effect strongly support the use of radiotherapy for the treatment of metastatic disease. However, deeper understanding of the molecular mechanisms underlying the abscopal effect are required to best benefit a larger proportion of patients with metastasis. Several groups including ours, reported the involvement of wild-type (wt) p53 in radiation-induced abscopal effects, however very little is known on the role of wtp53 dependent molecular mechanisms.

**Methods:**

We investigated through in vivo and in vitro approaches how wtp53 orchestrates radiation-induced abscopal effects. Wtp53 bearing (A549) and p53-null (H1299) NSCLC lines were xenotransplanted in nude mice, and cultured in 2D monolayers and 3D tumor spheroids. Extracellular vesicles (EVs) were isolated from medium cell culture by ultracentrifugation protocol followed by Nanoparticle Tracking Analysis. Gene expression was evaluated by RT-Real Time, digital qRT-PCR, and dot blot technique. Protein levels were determined by immunohistochemistry, confocal anlysis, western blot techniques, and immunoassay.

**Results:**

We demonstrated that single high-dose irradiation (20 Gy) induces significant tumor growth inhibition in contralateral non-irradiated (NIR) A549 xenograft tumors but not in NIR p53-null H1299 or p53-silenced A549 (A549sh/p53) xenografts. We further demonstrates that irradiation of A549 cells in vitro induces a senescence-associated secretory phenotype (SASP) producing extracellular vesicles (EVs) expressing CD63 and carrying DNA:RNA hybrids and LINE-1 retrotransposon. IR-A549 EVs also hamper the colony-forming capability of recipient NIR A549 cells, induce senescent phenotype, nuclear expression of DNA:RNA hybrids, and M1 macrophage polarization.

**Conclusions:**

In our models, we demonstrate that high radiation dose in wtp53 tumors induce the onset of SASP and secretion of CD63+ EVs loaded with DNA:RNA hybrids and LINE-1 retrotransposons that convey senescence messages out of the irradiation field triggering abscopal effect in NIR tumors.

**Supplementary Information:**

The online version contains supplementary material available at 10.1186/s13046-021-01883-0.

## Background

Radiotherapy (RT) represents a pivotal treatment for early and metastatic cancer. It is estimated that over half of all cancer patients can benefit from RT in combination with surgery or chemotherapy for disease management [1, 2]. Local RT exerts its clinical effects within the irradiated (IR) field for locoregional tumor control. However, noteworthy, regression in metastatic lesions distant from IR field, albeit uncommon, has been described in patients with different types of cancer including non-small cell lung cancer (NSCLC) [3–7]. This phenomenon, first described in 1953 [8], named “abscopal effect” (AE) has been considered for many years an enigma for the scientific community. A growing body of evidence sustains the immune system activation as the dominant player in radiation-induced AE. Indeed, it has been well-established that the release of a number of moieties endowed with immunostimulatory properties, released from irradiated lesions in the tumor microenvironment and systemic circulation, are capable of conveying death messages (apoptotic and / or necrotic signals) inducing the immunogenic cell death (ICD) [9, 10]. In particular, these molecules, also known as damage-associated molecular patterns (DAMPs), promote and convert the IR site into an immunogenic hub through the innate and adaptive immune response [11].

Very little is known about the molecular mechanisms involved in the AE. Camphausen et al. in 2003 [12] was the first study to link functional p53 with radiation-induced AE in mice. Accordingly, we following described AE in nude mice xenografted with wild type p53 (wtp53) colon cancer lines and receiving at least 20 Gy [13].

Numerous cancer cell lines carrying wtp53 develop hallmarks of senescence in response to radiation or to other DNA-damaging drugs [9, 14, 15], also known as therapy-induced senescence (TIS). TIS is highly dependent on wtp53 and p16INK4A pathways [16, 17] and is often associated with the nuclear DNA damage response (DDR) signalling structures called DNA-SCARS [18]. Recent literature has shed light on the importance of cytoplasmic nucleic acid sensors in DNA-damage response [19, 20]. Indeed, when DNA is damaged, the misplaced nucleic acids into the cytoplasm engage evolutionary-conserved sensors that trigger inflammation, IFNɑ/β pathways and, as recently hypothesized, the establishment of senescence-associated pro-inflammatory secretome [21]. Intriguingly, it was also reported that DNA:RNA hybrids structures may be largely constituted by transposable elements, in particular, long interspersed element-1 (LINE-1), the most ubiquitous transposable element in the mammalian genome, and proposed as hallmark of aging [22].

In the present work we demonstrated that single high dose irradiation (20 Gy) induces significant tumor growth inhibition in contralateral non-irradiated (NIR) A549 but not in NIRp53-null H1299 or p53 silenced A549 (A549sh/p53) xenografts. Moreover, we provided evidences that in vitro irradiated (IR) A549 cells adopt a senescence-associated secretory phenotype (SASP) secreting CD63 positive extracellular vesicles (CD63 + EVs) loaded with DNA:RNA hybrids and LINE-1 retrotransposon inducing the senescence of distant not irradiated cells.

On the basis of these data, we hypothesized that high RT doses induce AE based on the presence of functional p53 which triggers a senescence program in cancer cells with release of senescence-inducing molecules in the tumor microenvironment and blood circulation. Our data provide proof-of-concept of a novel molecular mechanism involved in the radiation-induced AE, which could help to improve the therapeutic outcomes of patients with advanced tumor disease harboring functional p53.

## Methods

### Cells

A549 cells were cultured in F12K (ATCC) medium; H1299 and THP-1 (ATCC Cat# TIB-202, RRID:CVCL_0006) cells were cultured in RPMI 1640 medium; RAW 264.7 cells (ATCC Cat# TIB-71, RRID: CVCL_0493) were maintained in DMEM. All media were supplemented with 10% FBS (Euroclone, Milan, Italy) and glutamine (2 mM) (Euroclone).

### Generation of A549sh/sip53 cells

Cells (3.0 × 10^4^ cells/6-wells plate) were infected with either lentiviruses LV-THM-sh/scr (scrambled sh-RNA, control) or LV-THM-sh/p53 at MOI 10 TU/cell, as described [23]. Early passages A549sh/p53 and A549sh/scr sublines were used in all experiments.

### Generation of H1299^p53+^ cells

Cells (3.0 × 10^4^ cells/6-wells plate) were transfected with p53wt expressing vector [24] (1 μg/well) with TransIT®-LT1 Transfection Reagent (Mirus, Thermo Fisher Scientific, Milan, Italy) following the manufacturer’s guidelines. Early transfected cells (72 h after transfection) were used in all the experiments.

### Mice and IR treatments

Xenograft tumours were generated in 40-days old athymic female nude mice (nu/nu CDl, Charles River Laboratories, Italy). as previously reported [13], and IR procedures detailed in Supplemental Methods. All experiments complied with regulations and ethics guidelines of the Italian Ministry of Health and were approved by the Institutional Animal Care and Use Committee of the Regina Elena Institute (Protocol n° 366/2015-PR del 08/05/2015).

### Immunohistochemical analysis

Consecutive 4-μm-thick tumor sections of formalin-fixed/paraffin-embedded xenografts were stained for Hematoxylin & Eosin (H&E), Lipofuscin detection or macrophage infiltration, as reported [25] and detailed in Supplementary notes.

### IL6 detection

Specimens from IR, NIR or UnIR A549 xenografts were accurately selected by a pathologist for areas including at least 50% of tumour cells, then scraped-off for RNA extraction. Digital qRT-PCR (dPCR) was performed to determine IL6 expression, as described in detail in the Supplementary Methods. IL6 quantification in the sera of IR and UnIR mice was determined with xMAP multiplex technology using a Human Magnetic Luminex assay (27-plex panel, Bio-Rad Laboratories, Segrate (MI) - Italy) as described in Supplemetary notes.

### In vitro IR experiments

A549, A549sh/p53 cells (as monolayer or 3D cultures), H1299, and H1299^p53+^ cells, were treated with different radiation doses (10 Gy and 20 Gy) using the linear accelerator Elekta Synergy Platform system (Elekta Oncology Systems, Stockholm, Sweden) as previously reported [26].

### MTS assay

Cytotoxicity was assayed using CellTiter 96® AQueous One Solution Cell Proliferation Assay (Promega, Milan, Italy) according to the manufacturer’s protocol and as described in Supplementary notes.

### Senescence assay

β-galactosidase staining was performed with the Senescence β-Galactosidase Staining Kit (Cell Signaling) on 2D and 3D cell cultures according to the manufacturer’s instructions and detailed in Supplementary notes.

### RNA extraction and qRT-PCR

Total RNA was extracted with TRIzol® Reagent (Invitrogen) followingmanufacturer’s instructions and qRT-PCR performed as described [27] and detailed in Supplementary Methods. The primer sequences used are listed in Supplemental Table 1.

### Retrotransposon PCR analysis

According to established protocols [22], purified poly(A) RNA, isolated from total RNA with NEBNext Poly(A) mRNA Magnetic Isolation Module (New England Biolabs, Euroclone), was analysed with Agilent 2100 Bioanalyzer (Agilent Technologies, Milan, Italy.) to assess yield and size distribution. Then, it was reverse-transcribed (10 ng) into cDNA with Taqman kit (Applied Biosystems Italia, Monza, Italy) by replacing random primer with a strand-specific primer1. RT-qPCR was performed using SYBR Green system (Bio-Rad Laboratories), and GAPDH adopted as the normalization control.

### Western blot analysis

Western Blot were performed as reported [27] and detailed in Supplementary Methods, with following mouse monoclonal primary antibodies: anti-p21WAF1 (NeoMarkers, Fremont, CA); anti-p53 (PAb 1801) (Invitrogen, Thermo Fisher Scientific Inc., Monza, Italy). The anti-vinculin (VLN01) was used as housekeeping (Invitrogen, Thermo Fisher Scientific Inc). Quantity One Software (Bio-rad Laboratories) was used for analyses.

### Confocal microscopy analysis

Cells were either fixed and permeabilized with ice-cold methanol or paraformaldehyde and incubated (overnight at 4 °C) respectively with primary anti-S9.6 antibody (1:100, Kerafast, Boston, MA, USA) or anti-CD63 antibody [MEM-259] (1:150, Abcam). Slides, after PBS washes, were incubated with the secondary goat anti-mouse Alexa Fluor 546 (Life Technologies) and confocal imaging performed with a Nikon A1 confocal laser scanning microscope.

### Foci analysis procedure

Confocal images were processed for DNA:RNA hybrids, CD63^+^EVs and ORF1p foci quantification. The foci segmentation procedure has been developed on purpose by both MATLAB and ImageJ built-in and self-implemented functions. Details are reported in Supplementary notes.

### EVs purification and characterisation

EVs used in present study were characterized according to the Minimal Information for Studies of Extracellular Vesicles guidelines, [28] as described in the Supplementary notes.

### RNA dot blotting

Isolated EV-RNAs (200 ng/μl) were spotted onto the Hybond-N+ membrane (GE Healthcare) optimized for nucleic acid transfer. For DNA:RNA detection, membrane were UV-cross-linked andupon blocking incubated with S9.6 primary antibody (overnight at 4 °C, Kerafast), and goat anti-mouse IgG-HRP (1:5000, Santa Cruz Biotechnology Inc.) before detection by chemiluminescence with Clarity™ Western ECL Substrate (Bio-Rad Laboratories).

### Macrophage polarization

Murine RAW264.7 and human THP-1 macrophage cell line were activated to M0 with M-CSF (Miltenyi Biotec, Bologna, Italy) (20 ng/ml, 48 h) or phorbol 12-myristate 13-acetate (150 nM) (Merck, Rome, Italy) respectively before to be exposed to isolated EVs The expression of M1/M2 polarization-associated markers were analysed by RT-PCR, as detailed in Supplementary notes.

### Statistical analysis

All experiments were performed at least three times, and quantifiable data derived from three independent experiments and reported as mean and standard deviation. Statistical analysis for in vitro and in vivo experiments was carried out using GraphPad Prism 8 software (GraphPad Software, San Diego, CA, USA), by applying the Student t-test for 2-group comparisons. Differences were considered significant at *p* < 0.05. R-package was used in vivo experiments for 2-group comparisons or Kruskal-Wallis multiple comparison test as appropriate. Differences were considered statistically significant when *p* ≤ 0.05. The medians of foci intensity distributions were tested with a) one-sample Wilcoxon signed-rank test; b) unpaired two-sample Wilcoxon-Mann-Whitney rank test; c) unbalanced two-way ANOVA. For all the tests *p*-value ≤0.01 was considered for statistical significance.

## Results

### Wtp53 induces abscopal effect in A549 xenografts

**Fig. 1:**
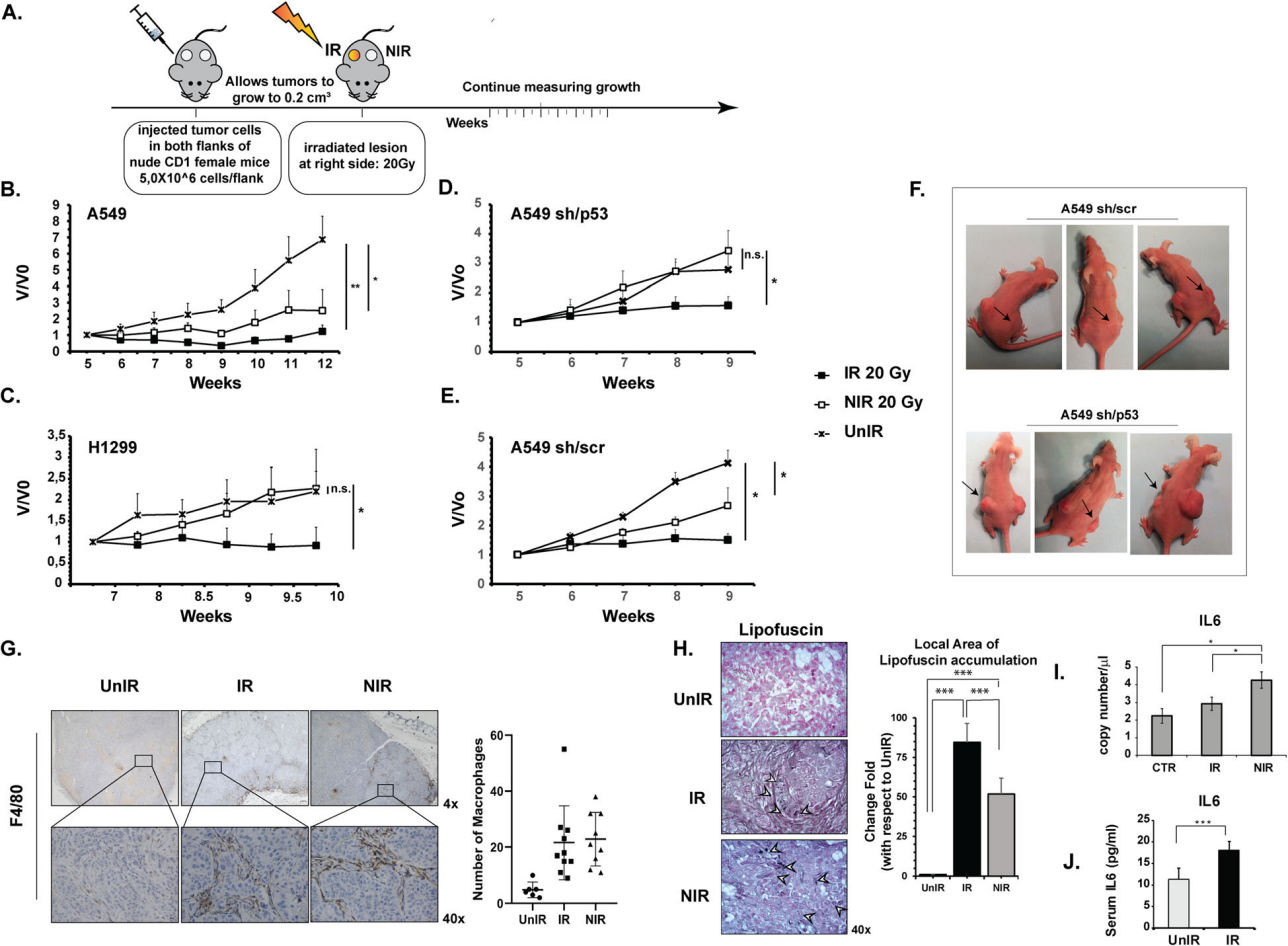
Wtp53 and high-dose delivery contribute to the Abscopal effect in in vivo NSCLC tumor models. **a** Experimental design of in vivo experiments aimed at evaluating the tumor-growth of irradiated (IR) and non-irradiated contralateral (NIR) tumors; control (UnIR) non-irradiated mice are also shown. **b** WTp53 A549 xenografts and (**c**) p53-null H1299 xenografts. Wtp53-depleted (sh/p53) (**d**) and control (sh/scr) (**e**) A549 xenograft tumor growth after RT are reported. Volumetric data were obtained using 8 mice/group and were normalized to the initial volume of tumor-bearing mice at the time of radiation (V0). Data represent the mean (± standard deviation, SD) of two independent experiments. (* *p* < 0.05; **p < 0.01). **f** Representative images of the abscopal effect in mice bearing functional p53 (A549 sh/scr). The mice bearing silenced p53 (A549 sh/p53) showed, as expected, tumor growth in the NIR contralateral mass. All the animals were xenografted subcutaneously on both flanks. Black arrows show tumors directly irradiated (20 Gy). **g** Immunohistochemical staining for F4/80 in mouse A549 xenograft tissue section. Images are representative of control (UnIR), 20 Gy-irradiated (IR) and contralateral non-irradiated (NIR) tumor masses infiltrated by F4/80-positive macrophages (arrow). The number of positive cells in 6 random field profiles was used for statistical analysis. **h** Lipofuscin staining of A549 xenograft tissue section to detect senescence cells. To quantify lipofuscin positive cells, a homemade Matlab tool was used. The appropriated mask needed to identify the lipofuscin positive cells in the investigated area was obtained using the function imageSegmenter as detailed in supplementary notes. (*** *p* < 0.0001) (**i**) IL6 expression through digital qRT-PCR analysis of RNA extracted from A549 xenograft tissue sections. **j** Different expression levels of IL6 in serum collected from A549 xenograft nude mice irradiated (IR) or not (UnIR) at 20 Gy. (* *p* < 0.05; ***p < 0.001)

To investigate wtp53 contribution in radiation-induced AE in vivo, an experimental procedure (Fig. 1a) was designed to mimic a real-world clinical setting, with directly irradiated (IR) xenograft lesion representing primary tumors and the contralateral untreated (NIR) representing metastases.

We observed AE in wtp53-bearing A549 (*p* < 0.05) (Fig. 1b) and sh/scr (*p* < 0.05) (Fig. 1e,f) NIR tumors, both of which showed significant tumor growth inhibition at 20 Gy-radiation dose. In contrast, accordingly with previous results [13], p53-null H1299 (Fig. 1c) and p53-depleted (sh/p53) A549 NIR tumors (Fig. 1d,f) showed no significant effects on tumor growth, as it almost overlapped that of the respective controls UnIR tumor group, thus confirming that functional p53 is required to trigger AE in vivo after high-dose radiation.

Notably, the AE only occurred when both tumor masses, IR and NIR, harboured functional p53, (Fig. S1) highlighting the central role of TP53 in cellular perception of the abscopal signal.

### Senescent cells, tumor-associated macrophages (TAM) and IL6 were detected in NIR A549 xenograft tumors

To microscopically investigate the RT-induced AE, A549 xenografts were RT treated/untreated, as in Fig. 1a, and tumors collected at various time-points after irradiation (days 1, 2, 4, 6, 10, 13) for histological examination. Extensive necrotic areas (data not shown) with significant infiltration of TAM (Fig. 1g) were found in IR and NIR xenografts tumors with respect to UnIR (*p* < 0.05) (Fig. 1g). TAM infiltrate was associated with the presence of senescent cells in both IR and NIR but not in UnIR tumor tissue sections, as shown by the increase of “age pigment” lipofuscin cytoplasmic accumulation (84,53 and 51,82 times higher in IR and NIR than in UnIR tumor sections, respectively, *p* < 0.0001) (Fig. 1h) and high mRNA levels of interleukin-6 (IL6), a cytokine released by senescent cells (Fig. 1i) (*p* < 0.05). Notably, significantly higher IL6 mRNA levels were found in NIR tumors with respect to IR tumors (Fig. 1i) (*p* < 0.05), and in accordance, higher human IL6 protein levels were detected in the sera of irradiated mice compared to UnIR group (Fig. 1j) (*p* < 0.001).

### Radiation treatment induces SASP in A549 cells in vitro

**Fig. 2:**
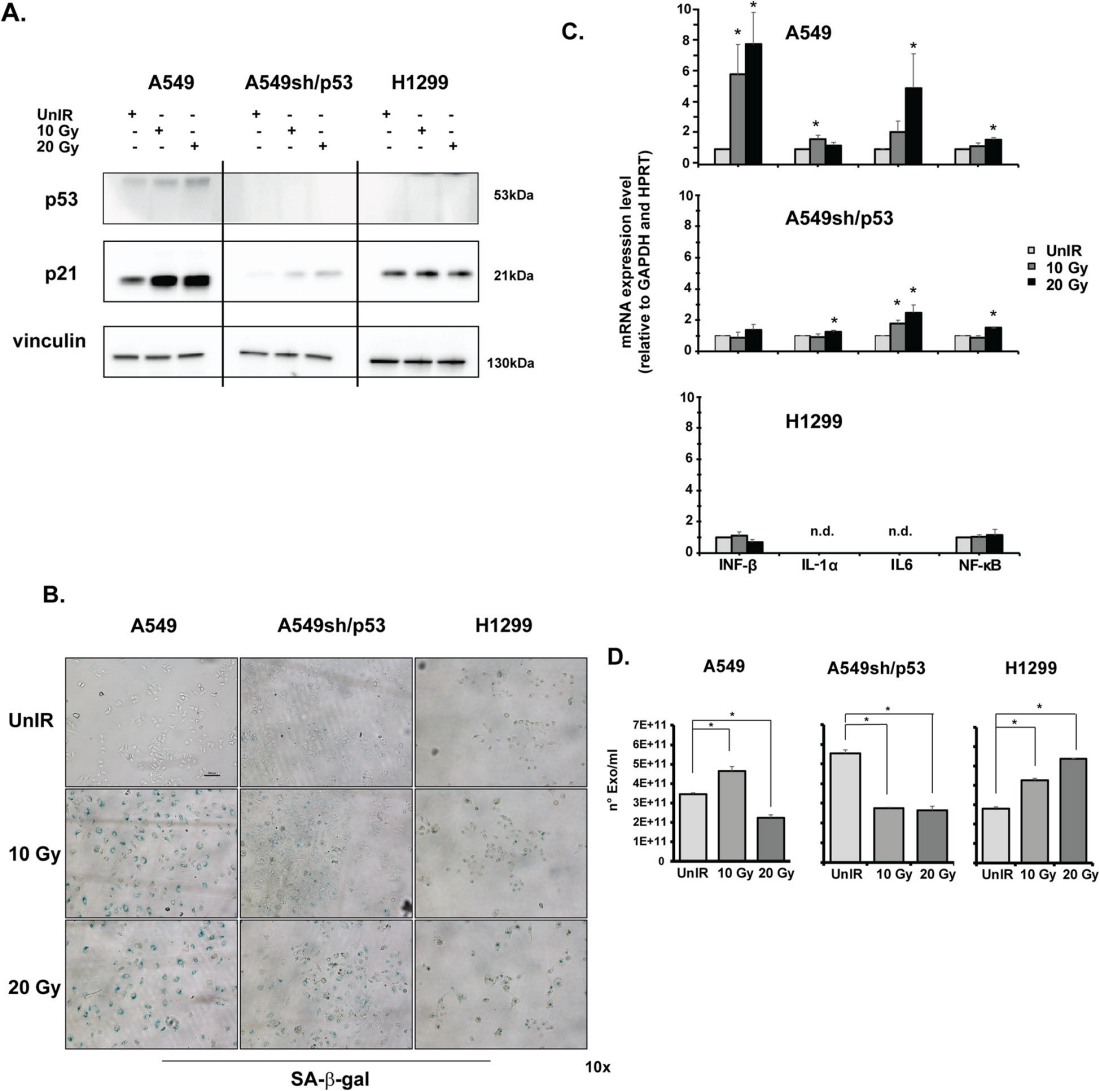
P53-dependent SASP induction after irradiation. **a** P53 and p21 expression in A549, A549sh/p53 and H1299 cells after different radiation doses detected by western blot. The image is representative at least of 2 independent experiments. **b** Representative images of senescence-associated β-galactosidase (SA-β-gal) staining detected after A549, A549sh/p53, and H1299 cell exposure to 10 or 20 Gy. Scale bar is 100 μm. **c** mRNA expression of SASP biomarkers using GAPDH and HPRT as housekeeping genes. Data are presented as mean ± SD. **d** EV quantification using Nanosight nanoparticle tracking analysis. EVs were isolated from the conditioned media of A549, A549sh/p53 and H1299 cells, as described in the Methods section (*p < 0.05)

To further investigate the radiation-induced senescence in our experimental models, senescence hallmark p21^Waf1/Cip1^ expression was evaluated in NSCLC cells treated with either 10 Gy or 20 Gy. Radiation treatments raised significantly the wtp53 protein levels and the expression of its target gene p21^Waf1/Cip1^ in A549 cells (Fig. 2a) consistently damped in A549sh/p53 cells. Conversely, no significant effects on p21^Waf1/Cip1^ expression were found upon radiation in p53-null H1299 cells (Fig. 2a). According to radiation-induced p21^Waf1/Cip1^ expression, a senescent phenotype with strong positivity for β-galactosidase (β-Gal) staining was revealed starting from 10 Gy in A549 cells (Fig. 2b). Conversely, positivity to β-Gal was significantly attenuated in A549sh/p53 and undetected in p53-null H1299 cells (Fig. 2b). Several markers closely related to SASP (IFN-β, IL-1α, IL6 and NF-kB, *p* < 0.05) were scored in A549 cells upon irradiation (Fig. 2c). The depletion of wtp53 (sh/p53) maintained the significant induction of IL-1α, IL6 and NF-kB markers albeit to a lesser extent with respect to parental A549 cells (Fig. 2c, *p* < 0.05). Conversely, none of these markers were induced in H1299 cells and, in particular, IL6 and IL-1α were not detectable (Fig. 2c).

**Fig. 3:**
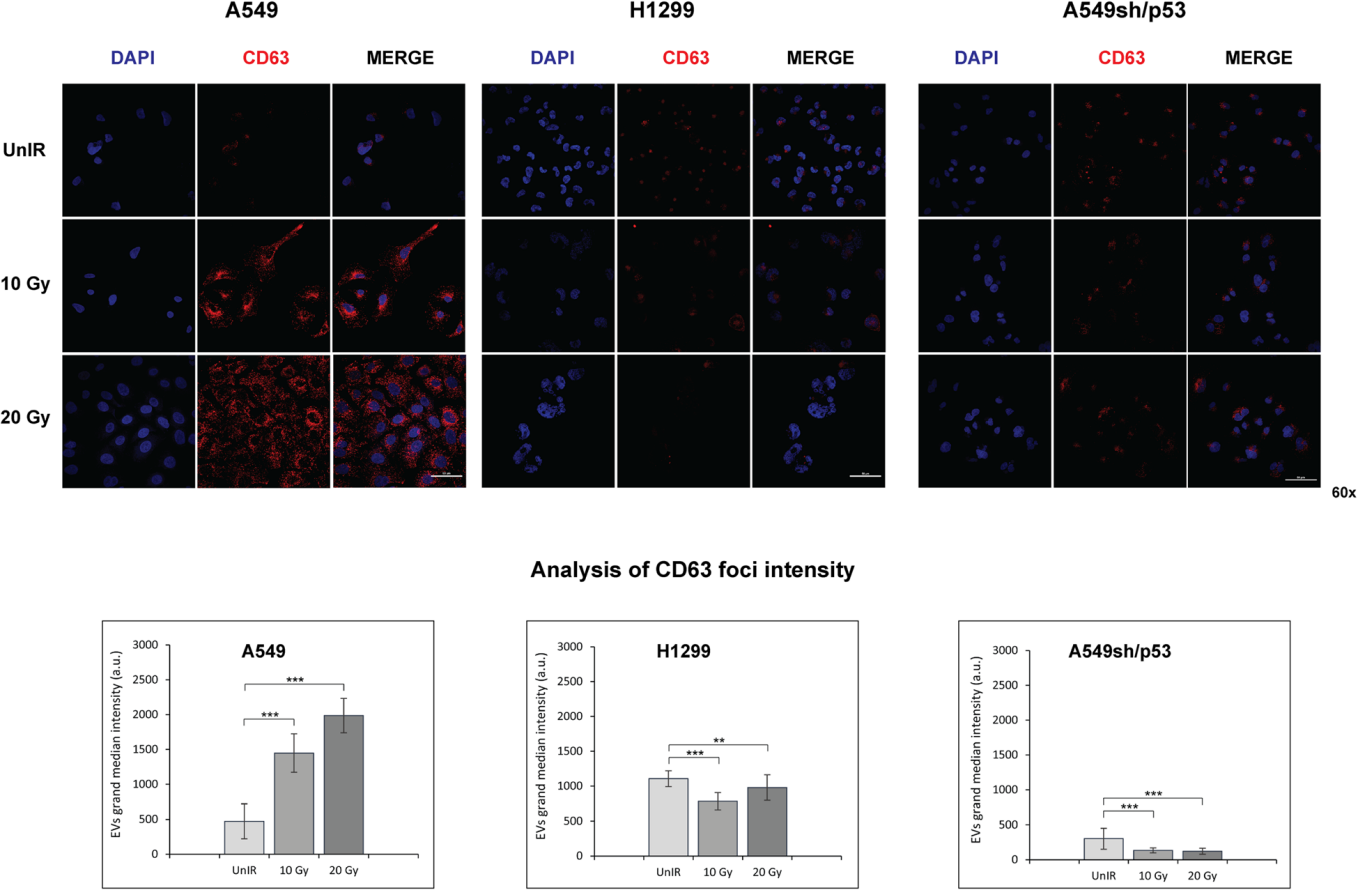
Immunofluorescence staining with CD63 antibody. The images are representative of A549, A549sh/p53 and H1299 cells exposed to different radiation doses. Fluorescent DAPI staining was used to visualize nuclear DNA. The images of the cells were captured by Nikon Eclipse Ti2 confocal microscope with 60x plan apochromatic oil immersion objective lens. Scale bars are 50 μm. Regional foci intensity quantification refers to the evaluation of CD63 expression in A549, A549sh/p53 or H1299 cells exposed to different radiation doses by confocal analysis. The effect of increasing irradiation doses on grand median intensities of nuclear and cytoplasmic foci of DNA:RNA hybrids. Data are presented as grand median ± MAD. (*p < 0.05, **p < 0.01 ***p < 0.001)

To investigate whether in our models the AE might occur through the SASP activation, we analysed the EVs released in the culture medium by IR A549, H1299 and A549sh/p53cells at 72 h post-radiation. The quantification analyses showed a significant increment in the number of EVs secreted from A549 cells and a decrease in p53-silenced cells (A549-sh/p53) (Fig. 2d). However, the significant radiation-dose-dependent increment of EVs production observed in H1299 cells (Fig. 2d) (*p* < 0.05), induced us to exclude the wtp53 involvement in modulating EVs secretion upon irradiation. Interestingly, confocal analysis revealed only in A549 cells a significant increase of CD63, an exosome marker, in terms of fluorescence intensity proportional to radiation doses (*p* < 0.001). The CD63-related signals significantly decreased in irradiated H1299, and accordingly to previous results, were significantly attenuated in A549sh/p53 cells, with no evident correlation with the radiation dose used (0.001 ≤ *p* < 0.01) (Fig. 3). All these data showed that wtp53 is require to induce SASP and biogenesis of a large amount of CD63 + EVs upon IR exposure.

### Radiation induces secretion of EVs carrying DNA:RNA hybrids in wtp53 NSCLC cell lines

**Fig. 4:**
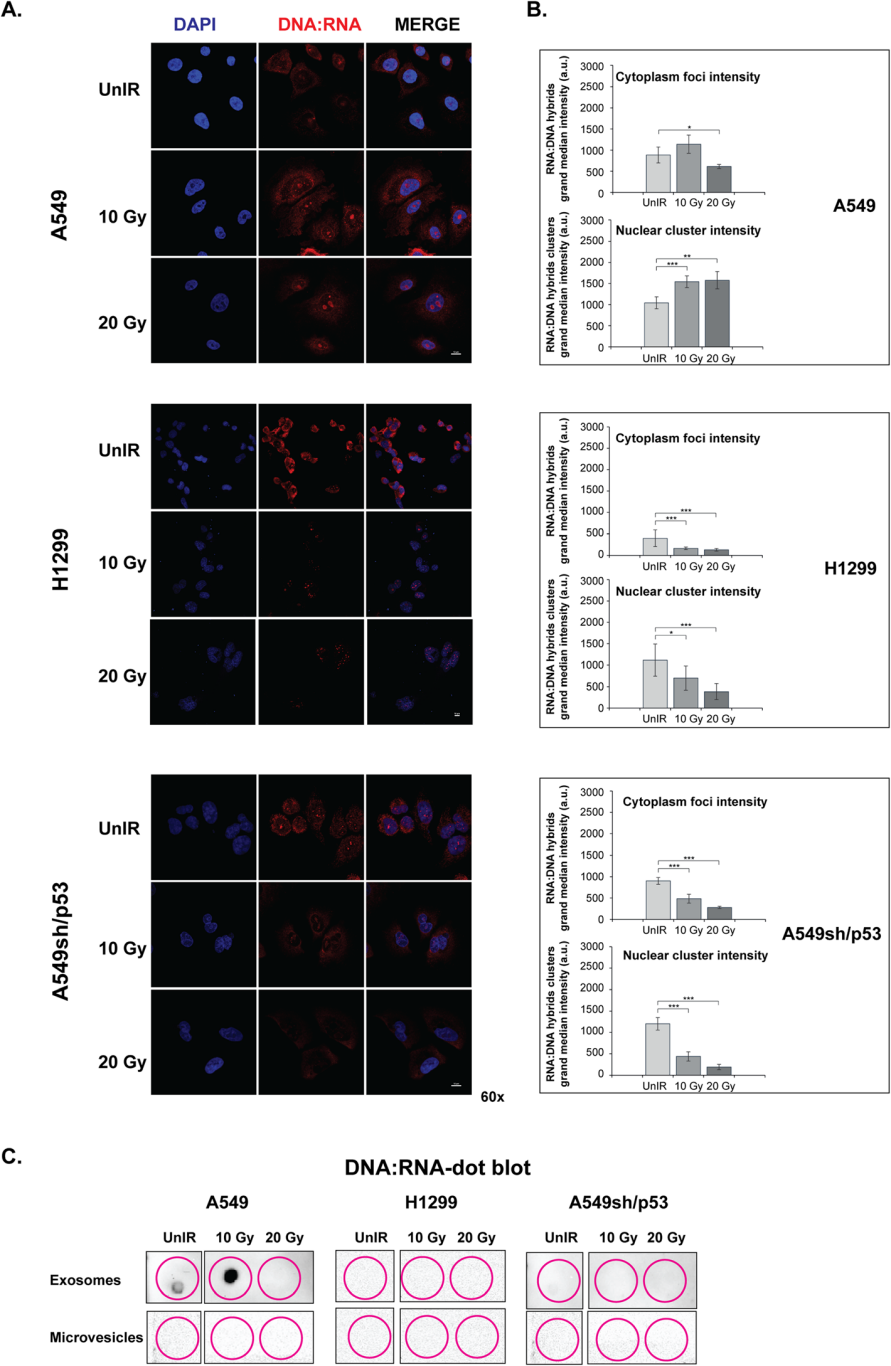
Cell irradiation induces the expression of DNA:RNA hybrid structures in NSCLC cell lines. **a** Immunofluorescence staining with S9.6 antibody. The images are representative of A549, A549sh/p53 and H1299 cells exposed to 10 or 20 Gy. Scale bars are 10 μm. **b** Regional foci intensity quantification. Effect of increasing irradiation doses on grand median intensities of nuclear and cytoplasmic foci of DNA:RNA hybrids. Data are presented as grand median ± MAD. **c** Dot blot analysis of RNA extracted from exosome or microvesicle fraction 72 h post-irradiation and immunoblotted with S9.6 antibody. (*p < 0.05, **p < 0.01 ***p < 0.001)

Since the cytoplasmic misplacement of nucleic acids has recently been suggested in the establishment of the senescence-associated pro-inflammatory secretome [21], we investigated the DNA:RNA hybrid structures upon irradiation. Interestingly, the DNA:RNA hybrid structures staining was significantly reduced in the cytoplasm of IR H1299 and IR A549sh/p53 cells (Fig. 4a, b, *p* < 0.001) but not in IR A549 cells where their still remarkable, albeit not significant, increase was observed at a dose of 10 Gy. Furthermore, radiation exposure induced significant accumulation of DNA:RNA hybrids in the nuclei of A549 cells that raise with the IR dose (0.001 ≤ *p* < 0.01) (Fig. 4b). Conversely, an opposite trend was observed in IR H1299 and IR A549sh/p53, with a significant decrease in both nuclear (0.001 ≤ *p* < 0.05) and cytoplasmic foci intensity (*p* < 0.001) (Fig. 4a, b).

To investigate whether the wtp53 dependent increase of DNA:RNA hybrids resulted in their secretion outside the cells, we investigated through dot blot analysis their presence in EVs isolated from conditioned media of A549, H1299, and A549sh/p53 cells after different radiation doses. The presence of DNA:RNA hybrids were detected in 10 Gy-IR A549 EVs but not in the microvesicle (MV) fraction. Conversely, neither EVs nor the MVs secreted by H1299 or A549sh/p53 were positive for the presence of DNA:RNA hybrids (Fig. 4c).

**Fig. 5:**
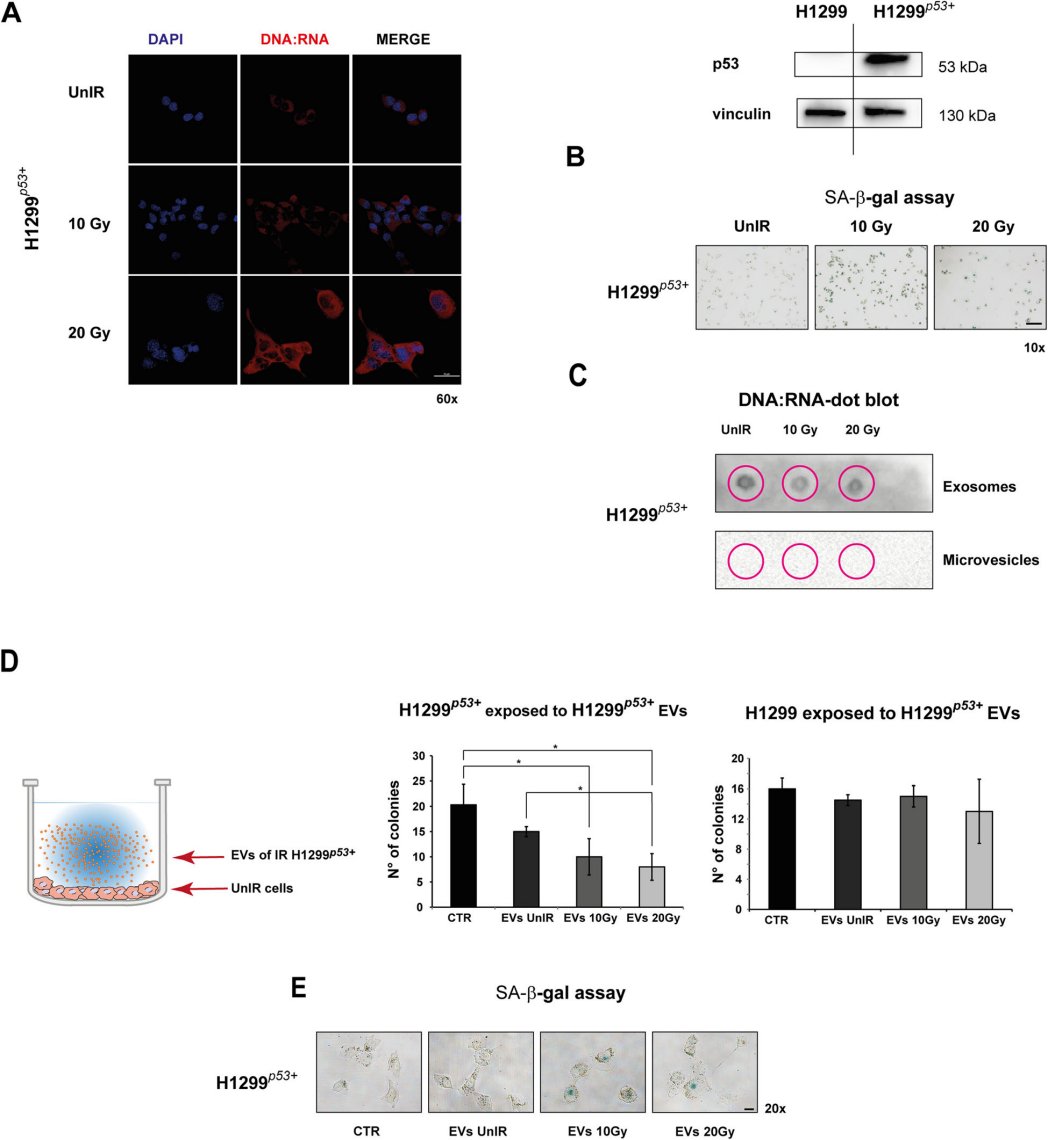
H1299 with forced wtp53 (H1299^p53+^) showed irradiation response similar to that observed in A549 cells. **a** H1299 cells with forced wtp53 showed a high level of DNA:RNA hybrid structures after irradiation. Cell images were captured by Nikon Eclipse Ti2 confocal microscope with 60x plan apochromat oil immersion objective lens. **b**. Representative images of senescence-associated β-galactosidase (SA-β-gal) staining detected after H1299^p53+^ cell exposure to 10 Gy or 20 Gy. Scale bar is 100 μm. **c** Dot blot analysis of RNA extracted from exosome or microvesicle fraction 72 h post-irradiation and immunoblotted with S9.6 antibody. **d** Colony-forming assay (CFA). Cells were exposed to EVs isolated from UnIR or IR H1299^p53+^ cells for 14 days. 0.05% crystal violet solution was used to visualize the generated colonies with more than 50 cells, which were quantified under inverted microscope (Olympus IX51 microscope, Olympus Corporation, Tokyo, Japan) by two independent observers. **e** Representative images of SA-β-gal staining of UnIR H1299^p53+^ cells after exposure to EVs isolated from culture medium of IR H1299^p53+^ cells

Furthermore, involvement of p53 in DNA:RNA hybrids secretion through EVs was also supported by additional experiments with H1299 cells forced to express transient ectopic wtp53 (H1299^p53+^) (Fig. 5). The irradiation exposure induced a cytoplasmic increase of DNA:RNA hybrids content, their appearance in secreted EVs, and a senescent phenotype in H1299^p53+^ cells similar to that observed in IR A549 cells (Fig. 5a-c). Accordingly, the EVs secreted by H1299^p53+^ carrying DNA:RNA hybrids also significantly inhibited the colony growth, and induced senescent phenotype of H1299^p53+^ (Fig. 5d,e).

**Fig. 6:**
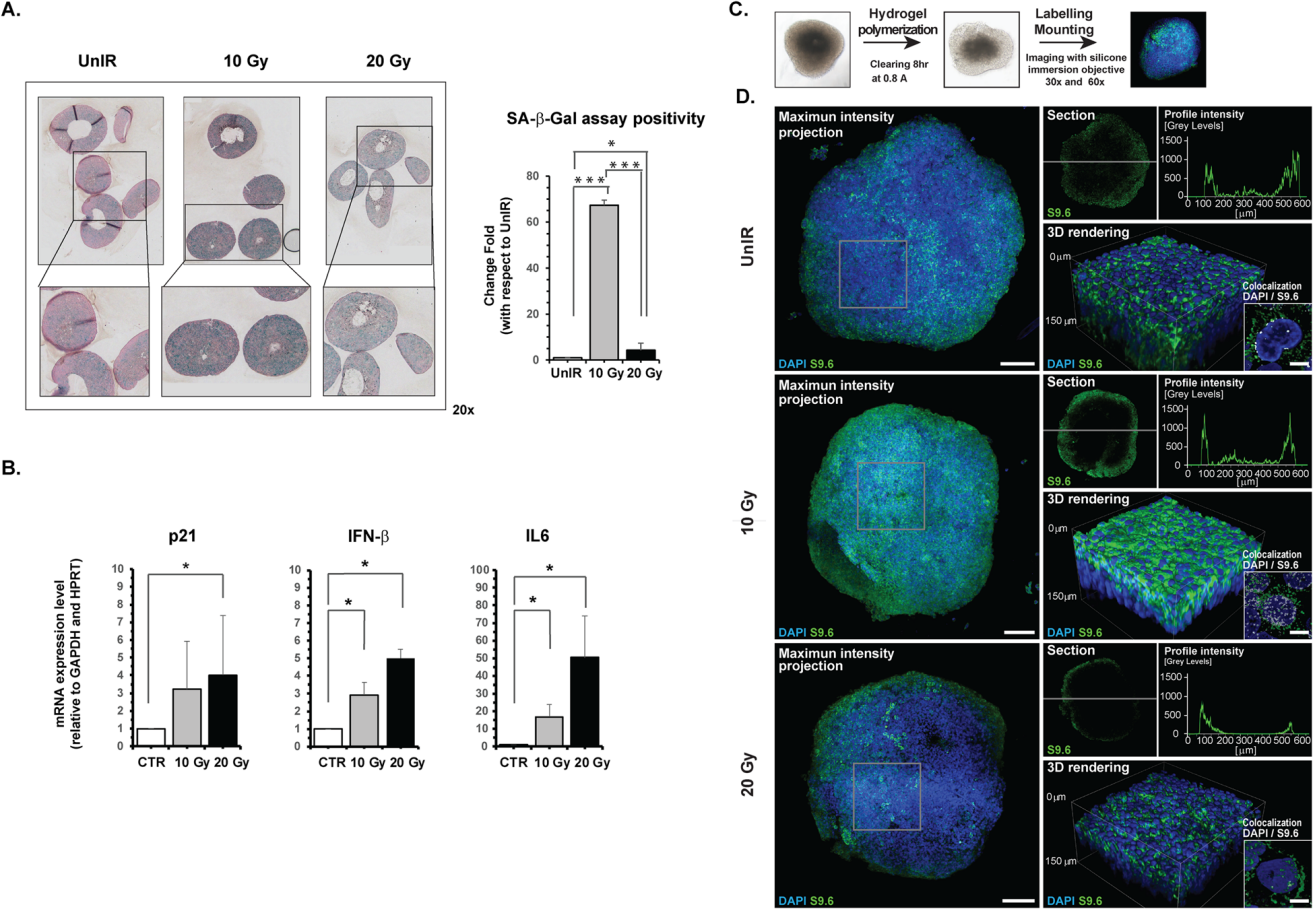
A549 cells grown as 3D models confirm the irradiation-induced SASP characterized by high synthesis of DNA:RNA hybrids structures. **a** Representative images of SA-β-gal staining performed on FFPE tissue sections of A549 spheroids fixed after exposure to 10 or 20 Gy. For evaluation of cells positive to SA-β-Gal assay, a homemade Matlab tool was used. The appropriated mask needed to identify the cells positive to SA-β-Gal assay in the investigated area was obtained using the function imageSegmenter; (* *p* < 0.05; *** *p* < 0.0001) (**b**) mRNA expression of SASP biomarkers detected in A549 cells grown as 3D spheroids using GAPDH and HPRT as housekeeping genes. Data are presented as mean ± SD. **c**, **d** Confocal immunofluorescence images representative of A549 spheroids after different irradiation doses showing cytoplasmic (green) and nuclear (white) localization of DNA:RNA hybrids (scale bar 10 μm). The micrograph shows the co-localization analysis performed through confocal high-resolution analysis. Bar graphs, mean ± error bars of fluorescent signals in the different compartments are also reported (see Methods section) (* *p* < 0.05)

Further studies carried out with more representative models of tumor architecture, such as three-dimensional (3D) cultures, confirmed the induction of a senescent phenotype (Fig. 6a,b). In particular, we observed a strong positivity to β-Gal assay upon irradiation of about 67 and 4 times higher in 10Gy-(*p* < 0.0001) and 20Gy-IR (*p* < 0.05) A549 spheroids, respectively, than in UnIR A549 3D culture (Fig. 6a). We also detected a significant induction of the SASP driver molecules p21^Waf1/Cip1^, INF-β, and IL6 in A549 spheroids, (Fig. 6b, *p* < 0.05). In addition, 3D Imaging analysis of optically cleared spheroids revealed a massive production of DNA:RNA hybrid structures after 10 Gy exposure and a substantial decrease in these structures in both the core and in periphery of cells after 20 Gy irradiation (Fig. 6c-d). Notably, confocal high-resolution analysis identified the nucleus as their site of production and confirmed their decrease after 20 Gy irradiation, the latter probably due to the secretion of the structures outside the spheroids (Fig. 6d). The same experiments performed on A549sh/p53 3D culture showed that irradiation did not induce SASP and the appearance of DNA:RNA hybrid structures, confirming what was observed in A549sh/p53 grown as a monolayer (Fig. S2A).

### IR A549 cells deliver abscopal signals through EVs loaded with DNA:RNA hybrids

**Fig. 7:**
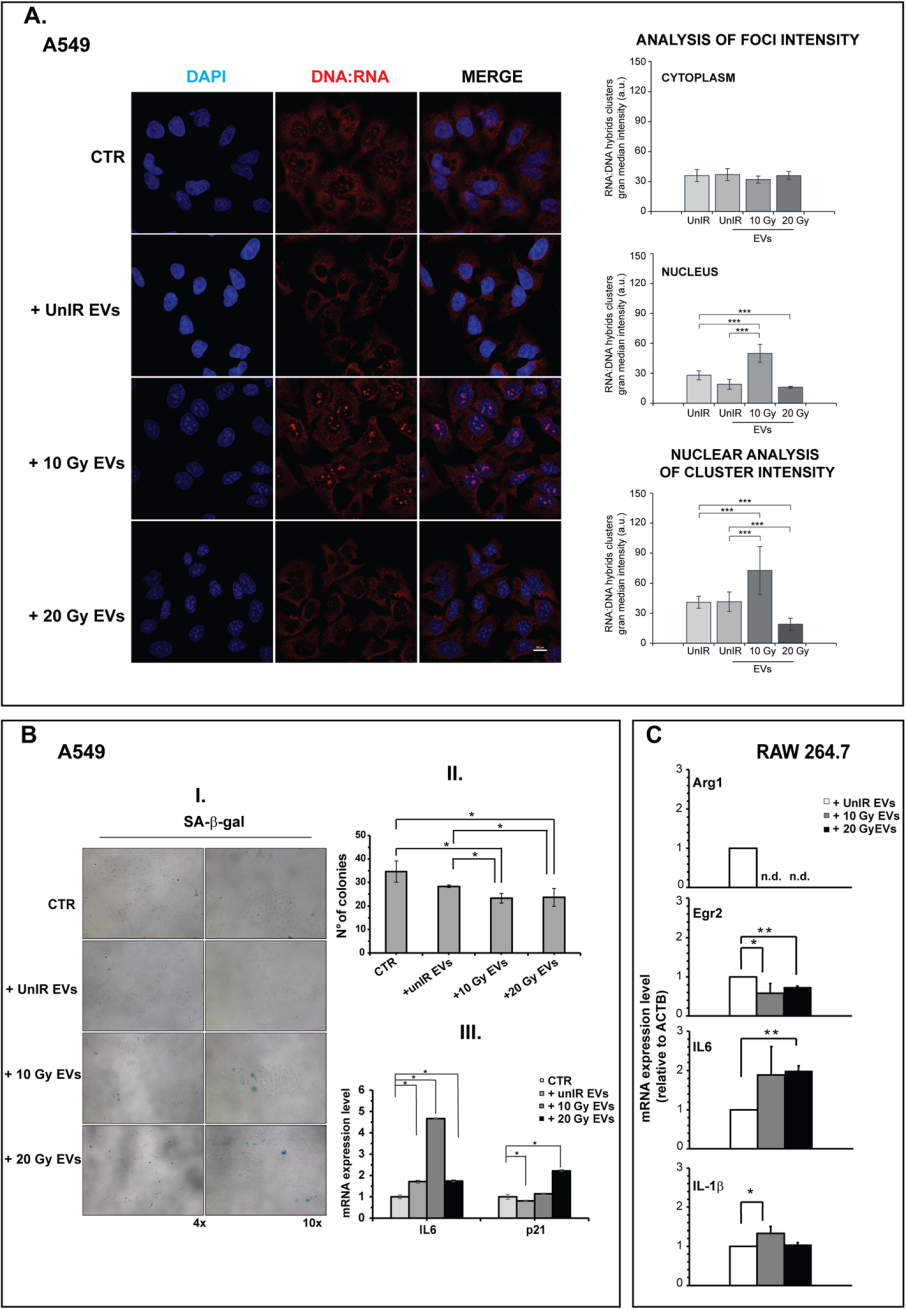
EVs secreted by high-dose irradiated A549 cells induce in vitro abscopal effects. **a** EVs secreted by irradiated A549 stimulate the synthesis of DNA:RNA hybrid structures. Immunofluorescence staining with S9.6 antibody. The images are representative of unIR A549 cells exposed to EVs isolated from culture medium of A549 cells non irradiated or irradiated at different doses. Cell images were captured by Nikon Eclipse Ti2 confocal microscope with 60x plan apochromat oil immersion objective lens. Data are presented as grand median ± MAD. **b** EVs secreted by irradiated A549 induce senescent phenotype. **(I)** Representative images of SA-β-gal staining of UnIR A549 cells after exposure to EVs isolated from culture medium of IR A549 cells. **II.** Colony-forming assay (CFA)**.** The cells were exposed to EVs isolated from UnIR or IR A549 cells for 14 days. 0.05% crystal violet solution was used to visualize the generated colonies with more than 50 cells, which were quantified under inverted microscope (Olympus IX51 microscope, Olympus Corporation, Tokyo, Japan) by two independent observers. **III.** Expression levels of senescence markers. p21, IL6 mRNA levels were measured by Real-Time PCR and normalized to GAPDH and HPRT-1. Data are the mean of two independent experiments. **(C)** EVs secreted by irradiated A549 induce M1 polarization in a murine macrophage cell line. Total RNA from RAW 264.7 M0 cells (see Methods section) exposed to EVs secreted by unirradiated, 10 Gy or 20 Gy-irradiated A549 cells were analyzed by RT-PCR for the expression of representative murine M2 genes (Arg1, Egr2) and M1/pro-inflammatory cytokines (IL-6, IL-1β). Expression data are given as fold increase over the mRNA level expressed by RAW 264.7 M0 exposed to UnIR EVs. Data are represented as mean ± SD of triplicate values of two independent experiments (*p < 0.05, **p < 0 .01 ***p < 0.001)

Confocal analysis revealed a significant increase in DNA:RNA hybrids in the nuclei of UnIR A549 cells exposed to 10Gy-IR A549 EVs (Fig. 7a, *p* < 0.001), but not when exposed to EVs from IR A549sh/p53 cells (Fig. S2B). Moreover, EVs from IR A549 (10Gy or 20 Gy) when placed in co-culture with UnIR A549 cells induced: i) β-Gal positivity (Fig. 7b-I) and significant inhibition of colony-forming ability of recipient cells (*p* < 0.05) (Fig. 7b-II); ii) increased expression of senescence markers p21Waf1/Cip1 and IL6, the latter specific for SASP (*p* < 0.05) (Fig. 7b-III).

Finally, according to in vivo results (Fig. 1g), the EVs from IR A549 cells induced the expression of pro-inflammatory cytokines and M1-associated genes in murine (RAW 264.7) and human (THP-1) established macrophage cell lines (Fig. 7c, Fig. S3). In particular, a significant increase in M1/pro-inflammatory cytokines (IL-1β, IL6) and reduction of M2-associated markers (Arg1, Egr2) were found in exposed RAW 264.7 cells (0.01 ≤ *p* < 0.05) (Fig. 7c). In parallel, a significant increase in the M1/pro-inflammatory markers (PPAR-α, TNF-α, STAT1 and IL-1β) was also observed in human THP-1 cells (*p* < 0.05) (Fig. S3).

### Loss of functional p53 induces high genotoxic damage upon radiation

To better characterize the genotoxic damage induced by high-dose radiation, we evaluated the presence of DNA single- (SSBs) or double-strand breaks (DSBs) in the nuclei of IR A549 and A549sh/p53 cells. In IR A549 cells comet assay revealed dose-related SSBs and DSBs induction, (Fig. S4A), and only a few micronuclei, a cellular marker of genomic instability [29, 30], at the highest radiation dose used. Conversely, IR A549sh/p53 cells presented DNA SSBs or DSBs only at the highest radiation dosage, albeit to a lesser extent than in A549 cells, and a strong induction of micronuclei at the lowest radiation dose (Fig. S4B). Notably, confocal analysis highlighted that the micronuclei formed after radiation exposure were not constituted by DNA:RNA hybrids (Fig. S4C). Finally, radiation induced strong inhibition in colony forming ability in both A549 and A549sh/p53 cells 12 days after dose exposure, albeit at 10 Gy-IR A549sh/p53 cells maintained a more significant colony-forming ability than A549 cells (Fig. S4D). These data combined with comet assay results, suggest that wtp53 sensitizes the IR A549 to apoptosis and that high percentage of DNA fragmentation probably mirrored the triggered programmed cell death.

### Irradiation induces the activation of LINE-1 retrotransposon in p53wt-bearing A549 cells

**Fig. 8:**
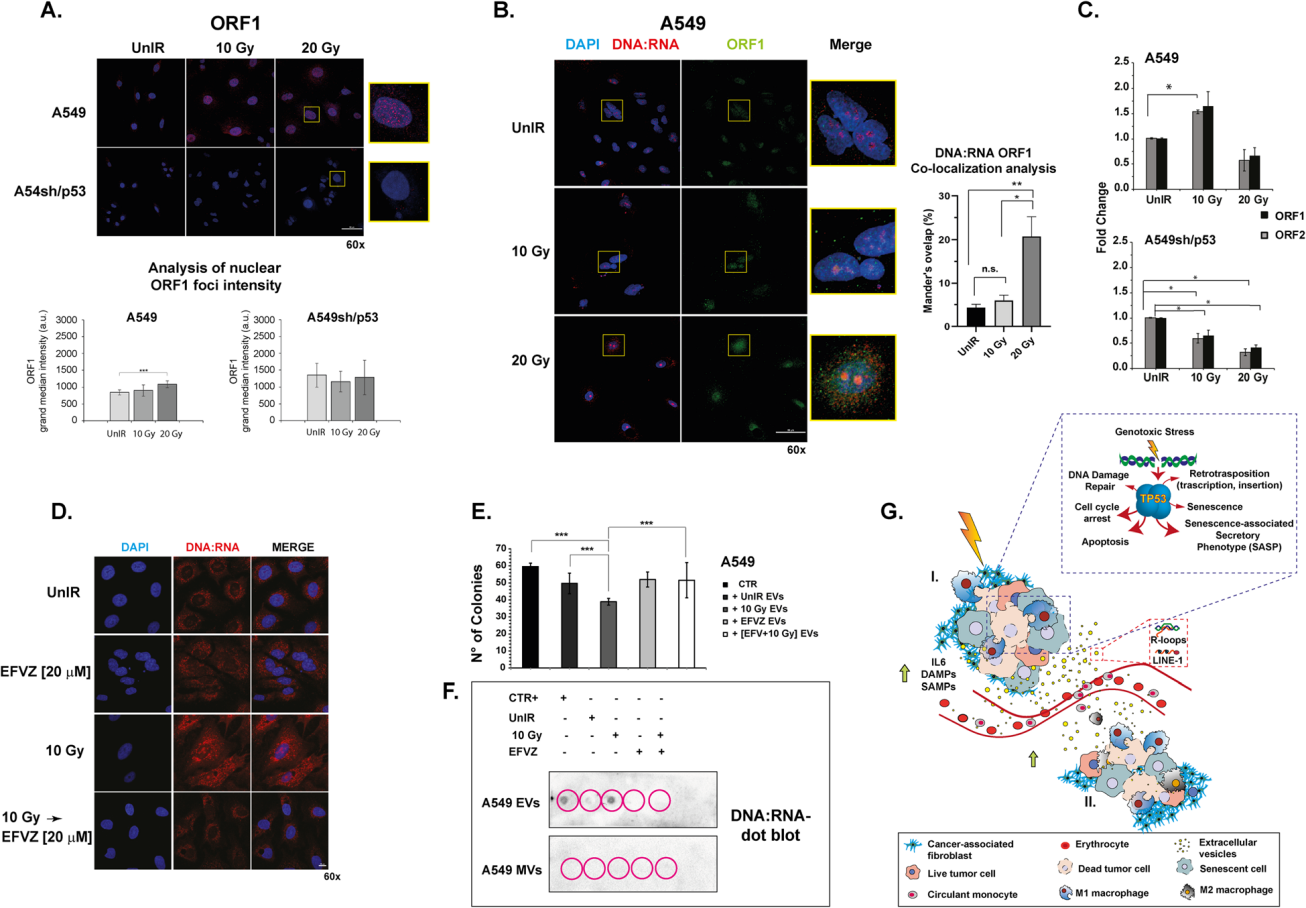
Correlation between DNA:RNA hybrids and retrotransposon ORF1. **a** Immunofluorescence staining with ORF1 antibody. The images are representative of A549, A549sh/p53 cells exposed to 10 or 20 Gy. Scale bar is 50 μm**.** Data are presented as grand median ± MAD. **b** DNA:RNA ORF1 co-localization analysis. Images are representative of A549 cells exposed to 10 Gy or 20 Gy. Scale bar is 50 μm. The average values of Mander’s ovelap (% of pixels that are overlapped, ± SEM) were calculated by software NIS-Element software’s tool (Nikon) and at least 20 cells were evaluated for each condition (*p < 0.05, **p < 0.01). **c** ORF1 and ORF2 mRNA expression in A549 and A549sh/p53 (see Methods section). Data are represented as mean ± SD of triplicate values of two independent experiments. **d** Representative confocal micrographs showing the positivity of A549 or A549sh/p53 cells to S9.6 antibody after exposure to Efavirenz (EFVZ) 20 μM, 10 Gy alone or followed by EFVZ 20 μM. Scale bar is 10 μm. (*p < 0.05, **p < 0.01 ***p < 0 .001). **e** Colony-forming assay (CFA). UnIR A549 cells were exposed to EVs isolated from unirradiated, irradiated, efavirenz-treated, and efavirenz-treated and irradiated A549 cells. 0.05% crystal violet solution was used to visualize the generated colonies with more than 50 cells, which were quantified under inverted microscope (Olympus) by two independent observers. **f** Dot blot analysis of RNA extracted from exosome or microvesicle fraction 72 h post-irradiation and immunoblotted with S9.6 antibody. The EVs and MVs were isolated from A549 cells exposed to different treatments. **g** Diagram illustrating the p53-mediated abscopal effect induced by radiotherapy. (**I)** Radiotherapy induces cell damage that leads to cell cycle arrest, necrosis, programmed cell death and cellular senescence. In particular, the cells bearing functional p53 may acquire SASP and release cytokines, inflammatory- (DAMPs) or senescence- (SAMPs) associated molecules. In addition, radiotherapy in the presence of wtp53 activates retrotransposon elements which, in turn, increase the level of genotoxic stress. SASP cells also release EVs conveying DNA:RNA hybrids and /or retrotransposon which, outside the field of irradiation (**II**), activate autodestruction mechanisms such as cellular senescence, apoptosis and innate immunity in p53-competent tumor cells

To explore the possibility that DNA:RNA hybrids may be constituted by LINE-1 retrotransposon, previously proposed as hallmark cellular senescence [22], we analyzed the expression level of both ORF-1 protein and ORF-1/ORF-2 mRNA in A549 and A549sh/p53 cells (Fig. 8a-c). Confocal analysis showed that both the cell lines expressed LINE-1, but only in IR A549 a significant increase in nuclear foci intensity was detected (*p* > 0.001) (Fig. 8a). Notably, the DNA:RNA ORF-1 co-localization analysis revealed an increase in Mander’s overlap (%) with increasing radiation doses in A549 cells, statistically significant at 20 Gy irradiation exposure (*p* > 0.001) (Fig. 8b), whereas an opposite trend was observed in IR A549sh/p53 (Fig. S2C). Furthermore, a significant increase in ORF1 and ORF2 mRNA (*p* < 0.05) levels was observed in IR A549 cells, but not in the corresponding IR A549sh/p53 cells (Fig. 8c). We also observed that efavirenz, an antiretroviral agent, reduced DNA:RNA hybrid expression in both IR and UnIR A549 cells (Fig. 8d). Accordingly, efavirenz pre-treatment abrogates the DNA:RNA hybrid structures occurrence in EVs IR A549 cells (Fig. 8f) which lose their inhibitory effects on colony-forming ability of UnIR A549 (Fig. 8e) (*p* < 0 .001). Overall, these data showed that DNA:RNA hybrids largely colocalized with the ORF1 signal were lost after treatment with an inhibitor of reverse transcriptase.

## Discussion

Over the years several hypotheses have been suggested to explain the molecular mechanisms behind the indirect anticancer effects of RT outside the radiation field. The difficulty in finding a single, identifiable reason for this occurrence highlights the complexity of AE and suggests that multiple pathways may contribute to triggering it. In this work, we showed that dysfunctional p53 hampers the activation of AE, suggesting only wtp53-positive cancer patients can benefit from this effect triggered by high radiation dose treatment. The presence of senescent cells and IL6 in contralateral NIR A549 tumor mass led us to investigate if the AE observed could be ascribed to the release of specific molecules from IR cells endowed with SASP.

To better clarify this phenomenon, we performed in vitro experiments aimed at emulating the in vivo irradiation condition. We showed that TP53 selectively regulates the secretion of CD63+ EVs carrying a senescence message composed of DNA:RNA hybrids and LINE-1 retrotransposons. Furthermore, the data obtained strongly support the hypothesis that CD63+ EVs together with cellular senescence, apoptosis and innate immunity may dictate the abscopal effect in NIR wtp53 A549 xenografts in nude mice. Of note, we observed that a radiation dose threshold of 10 Gy was needed for the abrogation of cytokines and the induction of DNA:RNA hybrid structures, as previously reported by Deng et al. [31]. Furthermore, we showed that high-radiation dose in concomitantly to SASP phenotype induced in wtp53 A549 cells a large amount of cytoplasmic expression of the tetraspanin CD63, mirroring the onset of an actively secreting phenotype. The onset of SASP was accompanied by the increase of DNA:RNA hybrids in nuclear and cytoplasmic cellular compartment and in EVs. In particular, only EVs isolated from IR wtp53 A549 or from IR H1299^p53+^, carried DNA:RNA hybrids that were not detectable in the EVs of p53-null H1299 or in p53-silenced A549 subjected to the same radiation treatment.

Furthermore, IR A549 or H1299^p53+^ EVs carrying the DNA:RNA hybrid cargo, when placed in contact with UnIR cells, induced a biomolecular make-up of recipient cells including the adoption of a senescent phenotype and reduced cell growth. These data strongly support that EVs secreted by IR cells with functional p53 are loaded with a “senescence” message.

To elucidate the factors governing the consistent macrophage recruitment observed in vivo, we exposed in vitro both murine (Fig. 7c) and human (Fig. S3) macrophage cell lines to EVs collected from conditioned media of high-dose-IR A549 cells. Results showed that only 10 Gy-IR A549 EVs cells, carrying a DNA:RNA hybrid, induced polarization of macrophages toward the M1 phenotype.

Finally, we explored the hypothesis that DNA:RNA hybrids may be constituted by transposable elements as previously reported for senescent cells by other authors [22]. It was previously reported that exposure to genotoxic stress such as irradiation often leads to the loss of global DNA methylation primarily from repetitive elements, in particular, LINE-1 [32]. The activation of LINE-1 elements may induce the synthesis of high amounts of DNA:RNA hybrid structures at the nuclear level, like those described in our work, mirroring the self-retrotranscription activity of retrotransposon elements that also require a cytoplasmic step before returning to the nucleus to complete their “reproductive cycle” [33]. Furthermore, Harris et al. [34] reported that a large number of p53-responsive elements or p53 DNA binding sites were detected in LINE-1 elements and that at least some were functional and served to increase LINE-1 mRNA expression levels. The authors also described the triggering of a positive feedback loop where p53 activated LINE-1 transcription, further stimulating the synthesis of ORF2p and causing more DSBs and more p53 activity, which amplified the amount of DNA damage and p53-mediated DDR. Sufficient DNA damage may result in the programmed destruction of cells, reducing LINE-1 transposition (considered as DAMP molecules) in somatic cells [34]. In our in vitro models we detected high ORF1 mRNA levels after irradiation. Furthermore, immunofluorescence experiments revealed a significant percentage of DNA:RNA ORF-1 colocalization, particularly evident in cell nuclei, which only increased in IR A549. Notably, we observed a dramatic reduction of DNA:RNA hybrids in IR A549 pre-treated with efavirenz, an anti-HIV drug that inhibits the expression of reverse transcriptase enzyme, essential for LINE-1 synthesis and its moving from one position in the genome to another via an RNA intermediate [35]. We also observed that efavirenz pre-treatment depleted DNA:RNA hybrids in of IR A549 EVs cells abrogating their inhibitory effects on colony-forming ability of UnIR cells. All these results strongly suggest that DNA:RNA hybrids conveyed by EVs secreted by IR cells with induced SASP are largely constituted by LINE-1 retrotransposon.

## Conclusion

The above observations, supported by our data, suggest that wtp53 activated by specific high radiation doses may induce the mobilization of transposon elements in both cell nuclei and cytoplasm, inducing a senescent phenotype. Functional p53 under genotoxic stress, as previously reported, plays a pivotal role in the establishment of SASP [36, 37] and in exosome secretion, which may affect adjacent cells and immune cells [14, 38, 39]. Our data suggest that p53 selectively regulates the secretion of CD63^+^ EVs carrying a senescence message composed of DNA:RNA hybrids and LINE-1 retrotransposons (senescence-associated molecular patterns, SAMPs) that can be perceived by cells outside the field of irradiation. This, in turn, may activate auto-destruction mechanisms such as cellular senescence, apoptosis and innate immunity (Fig. 8g). However, because EVs are exquisite carriers that can target specific cells [40] these data indeed suggest that the systemic delivery of the message may reach specifically cancer cells that belong to the same lineage as the primary tumor.

## Supplementary Information

**Additional file 1: Fig. S1.** WTP53 is required to trigger abscopal effects after irradiation. **Fig. S2.** A549sip53 cells grown as 3D models did not show irradiation-induced SASP; A549 cells grown as monolayer and exposed to IR A549sip53 EVs did not show expression of DNA:RNA hybrid structures; DNA:RNA ORF1 co-localization analysis in A549sip53 after irradiation exposure. **Fig. S3.** EVs secreted by irradiated A549 induce M1 polarization in a human macrophage cell line. **Fig. S4.** Analysis of DNA damage and growth rate in A549 and A549sip53 cells exposed to irradiation doses of 10 Gy or 20 Gy.

**Additional file 2.** Supplementary notes.

## Data Availability

All data generated or analysed during this study are included in this published article and its supplementary information files.

## Abbreviations

IR: Irradiated
UnIR: Unirradiated
NIR: Contra-lateral non-irradiated
RT: Radiotherapy
NSCLC: Non-small cell lung cancer
EVs: Extracellular vesicles
DAMPs: Damage-associated molecular patterns
DDR: DNA damage response
TAM: Tumor-associated macrophages
IL6: Interleukin-6
SASP: Senescence-associated secretory phenotype
SSBs: Single strand breaks
DSBs: Double strand breaks
SAMPs: Senescence-associated molecular patterns
