# Supplementary material for: TP53 drives abscopal effect by secretion of senescence-associated molecular signals in non-small cell lung cancer
Source: J Exp Clin Cancer Res. 2021 Mar 5;40:89. doi: 10.1186/s13046-021-01883-0 (PMC7934399; doi:10.1186/s13046-021-01883-0)
Supplement: Supplementary file 1 — Additional file 1: Fig. S1. WTP53 is required to trigger abscopal effects after irradiation. Fig. S2. A549sip53 cells grown as 3D models did not show irradiation-induced SASP; A549 cells grown as monolayer and exposed to IR A549sip53 EVs did not show expression of DNA:RNA hybrid structures; DNA:RNA ORF1 co-localization analysis in A549sip53 after irradiation exposure. Fig. S3. EVs secreted by irradiated A549 induce M1 polarization in a human macrophage cell line. Fig. S4. Analysis of DNA damage and growth rate in A549 and A549sip53 cells exposed to irradiation doses of 10 Gy or 20 Gy. [file 13046_2021_1883_MOESM1_ESM.pdf]

TP53 DRIVES ABSCOPAL EFFECT BY SECRETION OF SENESCENCE-ASSOCIATED MOLECULAR SIGNALS IN NON SMALL CELL LUNG CANCER

by Tesei A. et al.

SUPPLEMENTARY FIGURES.

Figure S1

A

IR shp53 *vs* scr

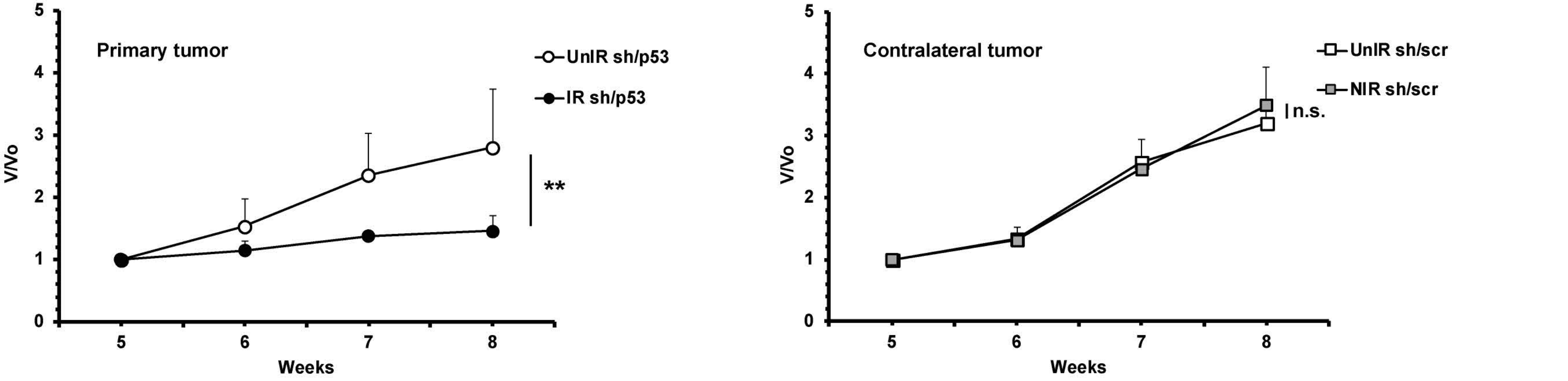

B

IR scr *vs* shp53

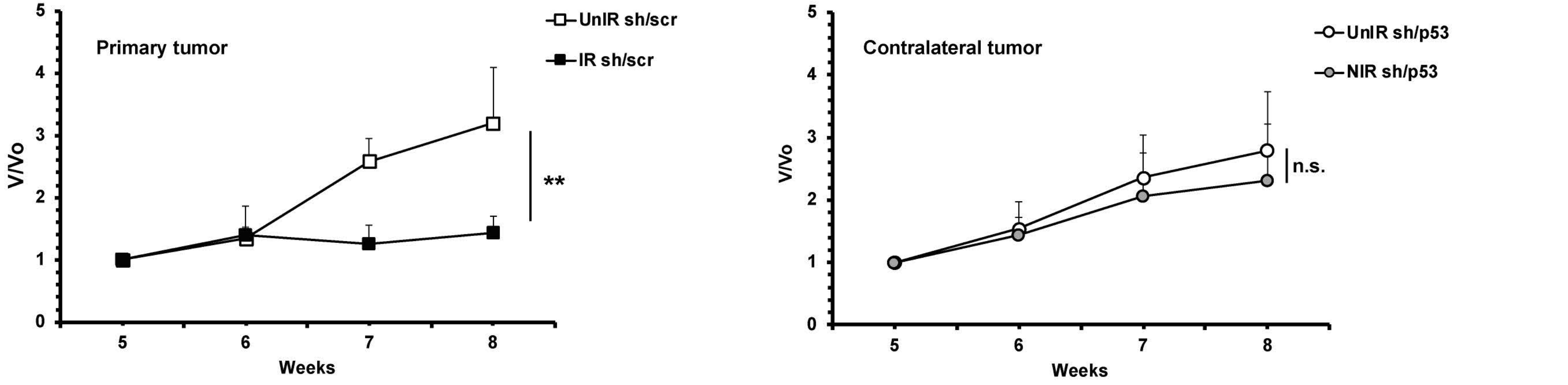

Figure S1. wtp53 is required to trigger abscopal effects after irradiation. Sh/scr and sh/p53 A549 sublines were both laterally s.c. implanted in each animal. Radiation dose (20 Gy) was delivered to sh/p53 (**A**) or sh/scr (**B**) tumors. Growth of IR, UnIR, and NIR tumors were determined by caliper measurements. Volumetric data were obtained using 8 mice/group and were normalized to the initial volume of tumor-bearing mice at the time of irradiation (V0). Data represent the mean (± standard deviation, SD) of two independent experiments. (**$p < 0.01$; n.s.= not statistically significant).

Figure S2

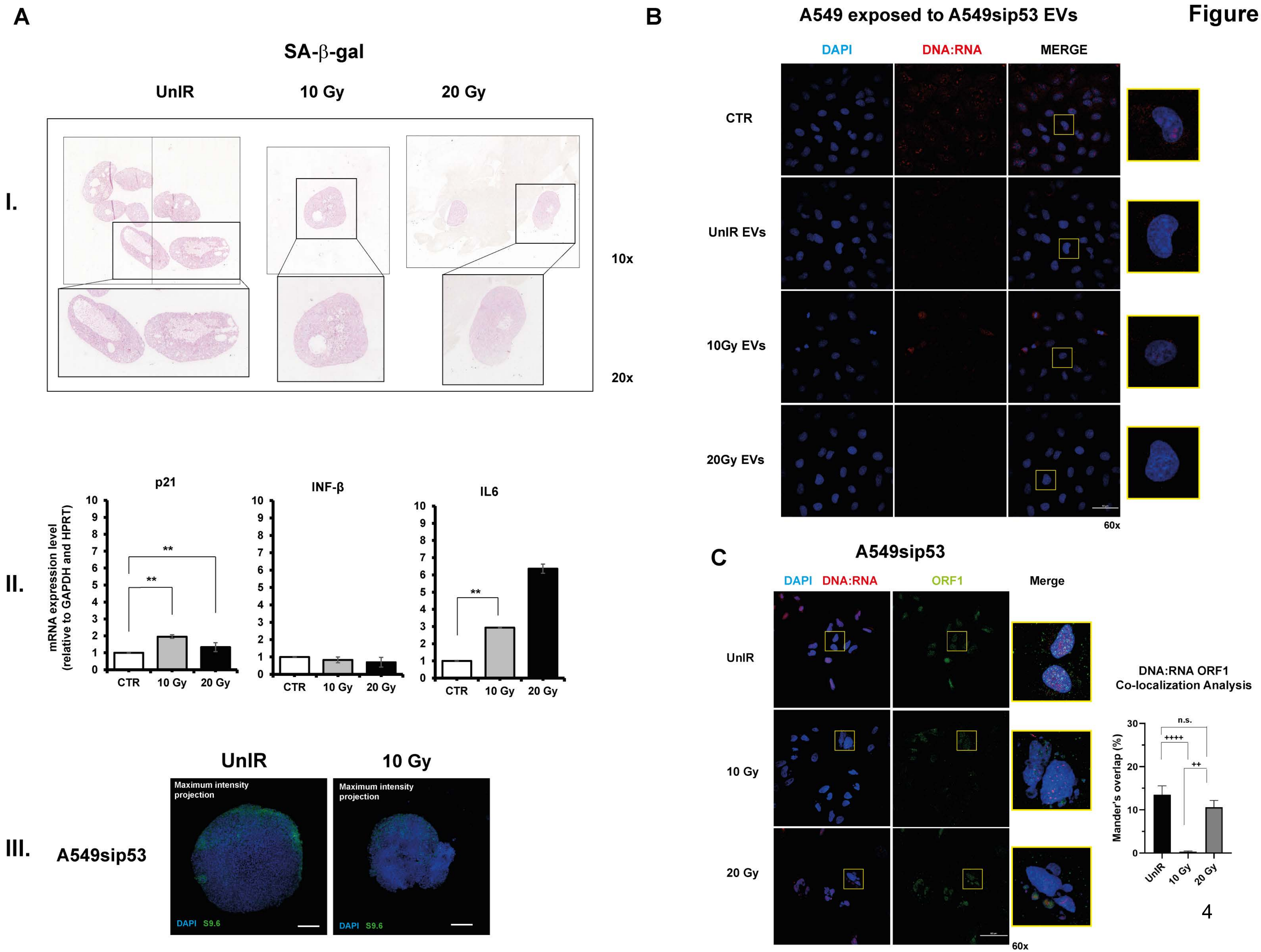
A
SA-β-gal
UnIR
10 Gy
20 Gy
I.
10x
20x
II.
p21
INF-β
IL6
mRNA expression level (relative to GAPDH and HPRT)
CTR
10 Gy
20 Gy
**
III.
A549sip53
Maximum intensity projection
DAPI S9.6
B
A549 exposed to A549sip53 EVs
DAPI
DNA:RNA
MERGE
CTR
UnIR EVs
10Gy EVs
20Gy EVs
60x
C
A549sip53
DAPI DNA:RNA
ORF1
Merge
UnIR
10 Gy
20 Gy
60x
DNA:RNA ORF1 Co-localization Analysis
Mander's overlap (%)
n.s.
++++
++

Figure S2. (**A**) A549sip53 cells grown as 3D models did not show irradiation-induced SASP (**I**) Representative images of SA-β-gal assay performed on FFPE tissue sections of A549sip53 spheroids fixed after exposure to 10 Gy or 20 Gy. The data showed absence of SA-β-gal staining. (**II**) mRNA expression of SASP biomarkers detected in A549sip53 cells grown as 3D spheroids using GAPDH and HPRT as housekeeping genes. Data are presented as mean ± SD. (**III)** Confocal immunofluorescence images representative of A549sip53 spheroids after irradiation at 10Gy. The data showed absence of nuclear (white) or cytoplasmic (green) DNA:RNA hybrids after radiation exposure. (scale bar 10 µm). (** $p<0.01$). (**B**) A549 cells grown as monolayer and exposed to IR A549sip53 EVs did not show expression of DNA:RNA hybrid structures. Cell images were captured by Nikon Eclipse Ti2 confocal microscope with 60x plan apochromat oil immersion objective lens. Scale bar is 50 µm. (**C**) DNA:RNA ORF1 co-localization analysis in A549sip53 after irradiation exposure. The images are representative of A549sip53 cells grown as monolayer and exposed to 10 Gy or 20 Gy. Scale bar is 50µm. The average values of Mander's ovelap (%, ± SEM) were calculated by software NIS-Element software's tool (Nikon) and at least 20 cells were evaluated for each condition (**$p < 0.01$, ****$p < 0.0001$).

Figure S3

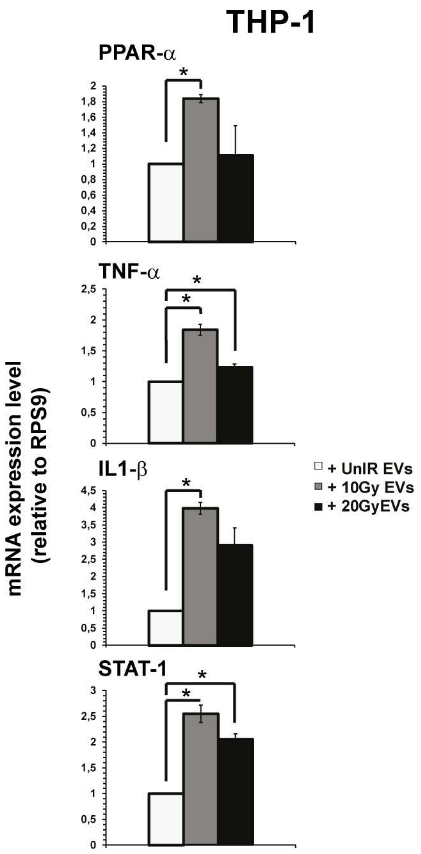

THP-1
PPAR-α
TNF-α
IL1-β
STAT-1
mRNA expression level
(relative to RPS9)
+ UnIR EVs
+ 10Gy EVs
+ 20GyEVs

Figure S3. EVs secreted by irradiated A549 induce M1 polarization in a human macrophage cell line. Total RNA from THP-1 M0 cells (see Methods section) exposed to EVs secreted by unirradiated, 10 Gy- or 20 Gy-irradiated A549 cells were analyzed by RT-PCR for the expression of representative M1 genes. Expression data are given as a fold increase over the mRNA level expressed by THP-1 M0 exposed to UnIR EVs. Data are reported as mean ± SD from triplicate values of three independent experiments (*p < 0.05, **p <0 .01).

A.

A549

UnIR | 10 Gy | 20 Gy

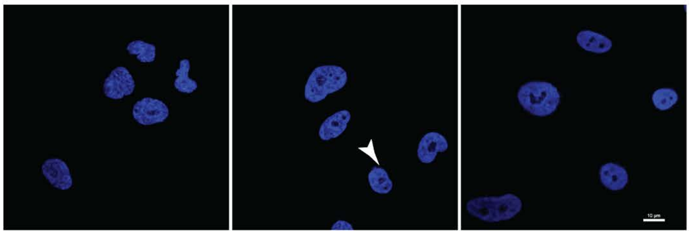

60x

% DNA in tail CLASS	UnIR		10 Gy		20 Gy	
	% DNA in tail (mean value)	n° nuclei	% DNA in tail (mean value)	n° nuclei	% DNA in tail (mean value)	n° nuclei
0 (<5%)	0.076±0.54	187	0.93±1.68	117	0.44±1.27	63
1 (5%-25%)	9.3±5.0	12	10.05±4.24	129	10.32±3.42	116
2 (25%-50%)	0	0	25.69±0.20	2	0	0
3 (50%-75%)	0	0	0	0	0	0
4 (>75%)	0	0	100±0	3	0	0

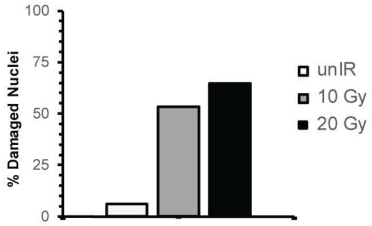

B.

A549sh/p53

UnIR | 10 Gy | 20 Gy

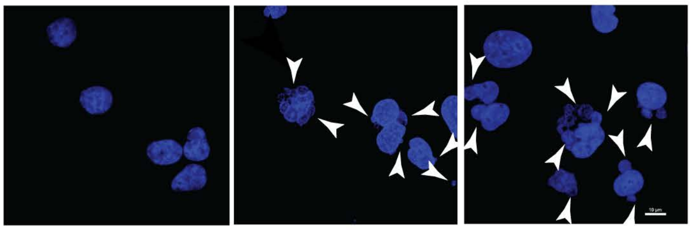

60x

% DNA in tail CLASS	UnIR		10 Gy		20 Gy	
	% DNA in tail (mean value)	n° nuclei	% DNA in tail (mean value)	n° nuclei	% DNA in tail (mean value)	n° nuclei
0 (<5%)	0.06±0.47	183	0.01±0.2	501	0.08±0.58	58
1 (5%-25%)	10.02±4.62	21	11.23±4.2	33	11.62±5.16	20
2 (25%-50%)	0	0	32.58±6.55	5	33.18±8.47	4
3 (50%-75%)	0	0	66.54±6.58	3	0	0
4 (>75%)	0	0	100±0	5	97.28±6.06	11

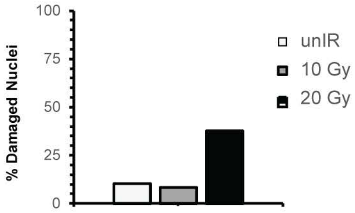

C.

DAPI | S9.6 | Merge

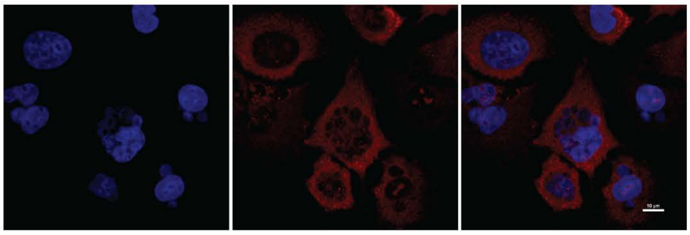

60x

D.

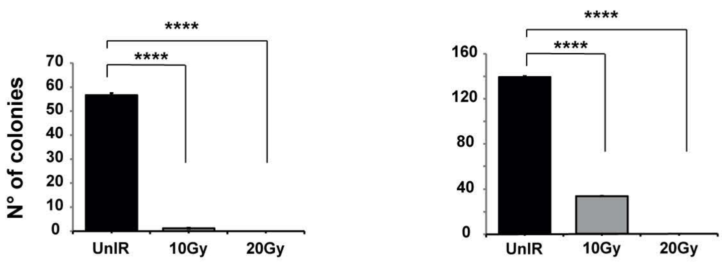

Figure S4. DNA damage features following exposure to radiation. (A) Evaluation of DNA damage in A549 **(A)** and A549sip53 **(B)** cells. Representative images showing micronuclei (white arrows) formation in A549 or A549sh/p53 cells after exposure to 10 Gy or 20 Gy; scale bars are 10 µm; fluorescent DAPI staining was used to visualize nuclear DNA. Cell images were captured by Nikon Eclipse Ti2 confocal microscope with 60x plan apochromat oil immersion objective lens. Single Cell Gel Electrophoresis assay (Comet assay) was performed to detect the DNA damage. Cells with damaged DNA (comets) were evaluated after 10 Gy or 20 Gy in A549 or A549sip53 cells. **(C)** Micrograph of A549sh/p53 irradiated at 20 Gy. Confocal images of micronuclei staining with S9.6 antibody. Scale bar is 10 µm. (**D**) Colony-forming assay. A549 and A549sip53 cells were exposed to irradiation doses of 10 Gy or 20 Gy. 0.05% crystal violet solution was used to visualize the generated colonies 12 days after irradiation.
