# Supplementary material for: TP53 drives abscopal effect by secretion of senescence-associated molecular signals in non-small cell lung cancer
Source: J Exp Clin Cancer Res. 2021 Mar 5;40:89. doi: 10.1186/s13046-021-01883-0 (PMC7934399; doi:10.1186/s13046-021-01883-0)
Supplement: Supplementary file 2 — Additional file 2. Supplementary notes. [file 13046_2021_1883_MOESM2_ESM.pdf]

TP53 DRIVES ABSCOPAL EFFECT BY SECRETION OF SENESCENCE-ASSOCIATED MOLECULAR SIGNALS IN NON SMALL CELL LUNG CANCER

by Anna Tesei et al.

SUPPLEMENTARY NOTES

Cell lines. A549, a cell line derived from primary Non-Small Cell Lung Cancer (NSCLC), and H1299, a NSCLC cell line derived from metastatic lesion (lymph node) from a patient who had received prior radiation therapy, were purchased from the American Type Culture Collection (ATCC, Rockville, MD; ATCC Cat# CCL-185, RRID:CVCL_0023; ATCC Cat# CRL-5803, RRID:CVCL_0060).

All the cell lines were checked periodically for mycoplasma contamination using the MycoAlert^{TM} Mycoplasma Detection Kit (Lonza, Milan, Italy). Both lines were expanded and maintained as a monolayer at 37 °C and subcultured weekly. The same culture media used for the monolayer cultures were used to grow the cells as 3D cell cultures.

Mice

In vivo irradiation experiments.

All experiments were performed in 40-days old athymic female nude mice (nu/nu CDl, Charles River Laboratories, Italy). Xenograft tumours were generated as previously reported [13]. Exponentially growing A549 and engineered sublines (sh/scr or sh/p53) (5.0 X 106/flank side) or H1299 p53-null (4.0 X 106/flank side) cells were subcutaneously injected into the right and left flanks of each animal. The number of cells to be injected was experimentally determined in order to obtain a comparable tumour volume (~ 0.2 cm^3) at the time of irradiation, about 3-4 weeks post-injection. For each experiment, tumour-bearing mice were divided into groups (n=8 mice/group) as follows: (i) mice with both tumours non-irradiated (UnIR); (ii) mice with one tumour irradiated

with 20Gy (IR 20Gy) and the contralateral non-irradiated (NIR 20Gy). Irradiation was performed using the Clinac 2100 C/D Varian with 6 MV photons, the dose rate was 2 Gy/min, while the shield used to mask the body outside the irradiated region was a combination of linac Tungsten jaws, Tungsten multileaf collimation and an additional shield of 4 cm of lead blocks placed on a box of 1 cm of PMMA (shielding more than 99.5% of the incident radiation). Before irradiation mice were anesthetized by intramuscular injection of 25 mg/kg Zoletil 50 (Virbac, LLDD-BP27-06511 Carros Cedex, France). Irradiation (20Gy/tumor) occurred within one hour for all experimental groups and performed as described previously [13]. Mice were placed in a polymethyl-methacrylate (PMMA) box above a bolus. The xenograft tumour was located away from organs at risk and kept fixed in the irradiation. In addition, the mice were shielded using a 5 mm PMMA plate and 2 mm of lead. The scattered dose was assessed using Gaf-chromic film, as described below. We performed in vivo dosimetry (IVD) at a specific point using Metal Oxide Silicon Field Effect Transistors (i.e. MOSFET, Best Medical Canada, Ottawa, Ontario) appropriately calibrated as previously reported53. Dose verification in a plan under the mouse was performed by using MD-V2-55 (International Specialty Product, Wayne, NJ, USA) and EBT2 Gaf-chromic film (International Specialty Product, Wayne, NJ, USA) appropriately calibrated as reported in Supplementary Materials. Both MD-V2-55 and EBT2 Gaf-chromic films were cut in 4 x 8cm2 pieces and placed under the mice during irradiation).

After irradiation, tumour-growth was monitored by calliper measurements twice a week, and tumour volumes [TV(cm3)] as estimated [13]. Animals were weighed weekly to assess any effects consequent to irradiation: no significant effects were observed with respect to the UnIR groups. Based on the experimental conditions, at specific time points animals were euthanized, blood samples collected and tumours excised. Sera were isolated and store at -80°C whereas tumors were formalin, fixed and paraffin embedded for histologic and immunohistochemical analyses.

In vivo Immunohistochemical assay: macrophage infiltration.

Three-to-four-mm-thick tissue sections were used for morphological evaluation after staining with Hematoxylin & Eosin (H&E) according to conventional histological procedures. H&E sections from 3 tumors for each group were scored by a trained operator for the determination of the percentage of tumor tissue occupied by necrotic areas. Infiltrating murine macrophages in tumour xenografts were detected using the anti-macrophage antibody ([RM0029-11H3] ab56297, abcam, Prodotti Gianni, Milan, Italy) as primary monoclonal antibody in indirect immunoperoxidase staining. Consecutive 4-µm-thick tissue sections derived from formalin-fixed and paraffin-embedded xenograft tumour samples were used as tissue substrates for immunohistochemistry. To count the macrophages, an image with an area of 0.64 mm^2 was created from six different visual fields. The number of positive cells in six random field profiles was used for subsequent statistical analysis. Under the various conditions, at least 6 images per section were analysed with 40x magnification. Macrophages were quantified in each image and areas of necrosis were excluded from the analysis. T-test was performed using the GraphPad Prism 8 software for statistical analysis.

Detection of Lipofuscin pigments. Lipofuscin, also known as an "age-pigment" (Terman A, and Brunk UT. Lipofuscin. Int J Biochem Cell Biol.2004; 36:1400-1404), is an aggregate of oxidized proteins that accumulate progressively, mainly in aged post-mitotic cells [Jung T et al. Lipofuscin: formation, distribution, and metabolic consequences. Ann N Y Acad Sci. 2007). It is considered a hallmark of aging and is also involved in the pathogenesis of certain age-related pathologies such as macular degeneration (Jung T et al.. Lipofuscin: formation, distribution, and metabolic consequences. Ann N Y Acad Sci. 2007; Kakimoto Y, et al. Myocardial lipofuscin accumulation in ageing and sudden cardiac death. Sci Rep 2019). Moreover, lipofuscin has an advantage over the traditional senescence marker b-galactosidase in that it is potentially usuable for FFPE archival tissue material**.** For the preparation of Sudan-Black-B (SBB) solution. 0.7 gr of SBB (Sigma-

Aldrich, Milan, Italy) was dissolved in 70% ethanol, covered with parafilm and thoroughly stirred overnight at room temperature. Filtered through filter paper and then filtered again through medium porosity frittered glass filter with suction. Throughout the process, it was important to avoid ethanol evaporation, which results in precipitation of the stain, so the solution was stored in an airtight container. Tissue sample sections were dewaxed with xylene and partially re-hydrated by passing the slides slowly through a series of decreasing concentrations of alcohols (from 100% to 70%). Subsequently the tissues are stained with a drop from freshly-prepared and filtered SBB solution (see Supplementary) for 5-7 minutes. Tissues were then washed first into 50% ethanol and then in distilled water. Finally tissues are counterstained with 0.1% Nuclear Fast Red (NFR) (Sigma-Aldrich) for 10 minutes at room temperature, washed and mounted into 40% Glycerol/TBS mounting medium. Lipofuscin staining was considered positive when perinuclear and cytoplasmic aggregates of blue-black granules were evident inside the cells. To quantify lipofuscin positive cells, we implemented a homemade Matlab tool. The appropriated mask needed to identify the lipofuscin positive cells in the investigated area was obtained using the function imageSegmenter. The following ranges [0-34], [0-58] and [0-144], were used in the channel R, G and B, respectively, for generating the mask for lipofuscin positive cells. The standard deviation of measurement in 10 randomly obtained subsets of investigated slide was calculated as standard deviation of measurement.

IL6 Detection in tumor tissue section through Digital qRT-PCR. Cells were lysed in 50 mmol/L KCl, 10 mmol/LTris-HCl pH 8.0, 2.5 mmol/L MgCl2, and Tween-20 0.45%, with the addition of Proteinase K at a concentration of 1.25 mg/mL, overnight at 56C. Proteinase K was inactivated at 95C for 10 minutes, after which samples were centrifuged twice to eliminate debris. Supernatant was assessed for RNA quality and quantity by Nanodrop (Celbio, Euroclone) and then underwent PCR amplification of IL6. All dPCR experiments were carried out using the chip-based QuantStudio^{TM} 3D Digital PCR system (Applied Biosystems). IL6 was run in singleplex using 10 ng cDNA. Reaction

mixes containing either cDNA or water (no-template controls) were first prepared by adding 2X QuantStudio 3D™ Digital PCR Master Mix v2 (Applied Biosystems) and 20X gene specific assay in a total volume of 15.5 µl. Chips were run using GeneAmp PCR System 9700 (Applied Biosystems) by applying the following conditions: hold at 96°C for 10 min; 45 cycles of 60°C for 2 min and 98°C for 30 sec; hold at 60°C for 2 min. At the end of the reaction, chips were processed using the QuantStudio™ 3D Digital PCR system (Applied Biosystems) and analyzed with QuantStudio™ 3D Analysis Suite™ software (version 3.0.3).

IN VITRO STUDIES

MTS Assay. Cytotoxicity was assayed using CellTiter 96® AQueous One Solution Cell Proliferation Assay (Promega, Milan, Italy) as previously described [ref] and described in Supplementary notes. Cells were seeded onto a 96-well plate at a density of 3×10^3 cells per well. Cell lines were exposed to scalar concentrations of the drug Efavirenz, ranging from 20 to 80µM. The effect of the drug was evaluated after 24, 48 and 72 h of continued exposure. Two independent experiments were performed in octuplicate. The optical density (OD) of treated and untreated cells was determined at a wavelength of 490 nm using a fluorescence plate reader. Dose response curves were created by Excel software. IC_{50} values were determined graphically from the plot.

β-galactosidase Senescence Assay on 2D and 3D cell cultures. β-galactosidase staining was performed with the Senescence β-Galactosidase Staining Kit (Cell Signaling) according to the manufacturer's instructions. Briefly, A549, A549sh/p53 as well as H1299 1.5x105 cells were plated in a 6-well plate after radiation treatment. After 3 days, cells were fixed in 1x fixative solution for 10 minutes at room temperature and stained overnight at 37°C with the β-galactosidase staining solution at pH 6.0. Images were acquired with an inverted Olympus IX51 microscope (Olympus) with the 4x and 10x objectives, equipped with a Nikon Digital Sight DS-Vi1 camera (CCD vision sensor, square pixels of 4.4 µM side length, 1600 × 1200 pixel resolution, 8-bit grey level) (Nikon Instruments, Spa. Florence, Italy). For 3D cell cultures, 3 days after radiation treatment, spheroids

of A549 were fixed in 1x fixative solution for 1-2 hours at room temperature and stained overnight at 37°C with the β-galactosidase staining solution at pH 6.0. Tumor spheroids were then fixed again in 10% buffered formalin for 20 hours and embedded in paraffin. Five-micrometre-thick sections of each sample were counterstained for nuclei with 0.1% Nuclear Fast Red (NFR) (Sigma-Aldrich) for 10 minutes at room temperature, washed and mounted into 40% Glycerol/TBS mounting medium. High-resolution whole slide images of stained spheroids were acquired using the MicroVisioneer Manual WSI system (MicroVisioneer, 20x magnification). To quantify cells positive to SA-β-Gal assay, we implemented a homemade Matlab tool. The appropriated mask needed to identify the cells positive to SA-β-Gal assay in the investigated area was obtained using the function imageSegmenter. The following ranges [81-174], [67-213] and [0-216], were used in the channel R, G and B, respectively, for generating the mask for SA-β-Gal assay positive cells. The standard deviation of measurement in 10 randomly obtained subsets of investigated slide was calculated as standard deviation of measurement.

Comet assay. In the present work we performed comet assay under alkaline conditions to detect both single and double strand breaks. The protocol, including the conditions for cell lysis and electrophoresis of comet assay, was carried out according to the manufacturer's guidelines and used the reagents provided in the assay kit (Comet assay, Trevigen, R&D Systems, Milan Italy). Briefly, after 72 hours from irradiation, $5x10^5$ cells were suspended in LMAgarose (at 37°C) at a ratio of 1:10 (v/v), and 75 µl were immediately transferred onto the comet slide. The slides were immerged for 1 hr at 4°C in a lysis solution, washed in the dark for 1 hr at 4°C in alkaline solution, then electrophoresed for 30 min at 20V. Slides were immerged twice in dH2O for 5 minutes each, then dipped in 70% ethanol and stained with 20 µl of diluted SYBR® Green Master Mix (Bio-rad Laboratories). The extent of DNA damage was evaluated quantitatively by EVOS FL microscope (Cell Imaging Systems, Thermo Fisher Scientific) by scoring at least 100 nucleoids of different categories, using the free software Cell Profiler (version 2.1.1). Percentage of DNA in tail for

different categories of comets was expressed, as previously described by O. Garcia et al. (Garcia O, Romero I, González JE, Mandina T. Measurements of DNA damage on silver stained comets using free Internet software. Mutat Res. 2007 Mar 5;627(2):186-90.)

RNA extraction and qRT-PCR quantification.

Briefly, RNA was quantified using the Nanodrop® ND-1000 spectrophotometer system and stored at -80°C. Reverse transcription reactions were performed in 20 µL of nuclease free water containing 200 ng of total RNA using iScript cDNA Synthesis kit (Bio-Rad Laboratories). Real-Time PCR was run using the 7500 Real-Time PCR system (Applied Biosystems). Reactions were carried out in triplicate at a final volume of 20 µL containing 20 ng of cDNA template, TaqMan universal PCR Master Mix (2X), and selected TaqMan assays (20x) (supplementary Table). Samples were maintained at 50°C for 2 minutes, then at 95°C for 10 minutes followed by 40 amplification cycles at 95°C for 15 seconds and at 60°C for 30 seconds. The amount of mRNA was normalized to the endogenous genes GAPDH and HPRT-1. Reference genes were chosen using the geNorm VBA applet for Microsoft Excel to determine the most stable reference genes. Data showed the average of triplicates ± standard deviation (SD) and were representative of three independent experiments.

Table S1. TaqMan gene expression assay.

**Gene Symbol**	**Assay ID**	**Amplicon (bp)**
IL1-alpha	Hs00174092_m1	69
IL1-1 beta	Hs00174097_m1	94
IL6	Hs00985639_m1	66
IFNβ1	Hs01077958_s1	73

TNF-alpha	Hs00174128_m1	80
NFKB1	Hs00765730_m1	66
GAPDH	Hs03929097_g1	58
HPRT1	Hs01003267_m1	72
STAT1	Hs01013996_m1	66
PPAR-alpha	Hs00947538_m1	118
RPS9	Hs00955300_m1	95
p21	Hs00355782_m1	66
IL6	Mm00446190_m1	78
IL-1b	Mm00434228_m1	90
Arg1	Mm00475988_m1	65
Egr2	Mm00456650_m1	73
ACTB	Mm00607939_s1	115

Western blot analysis

Briefly, cell proteins were extracted with M-PER Mammalian Protein Extraction Reagent (Thermo Fisher Scientific) supplemented with Halt Protease Phosphatase Inhibitor Cocktail (Thermo Fisher Scientific). Mini-PROTEATGX™ precast gels (4–20% and any kD) (BIO-RAD) were run using Mini-PROTEAN Tetra electrophoresis cells and then electroblotted by Trans-BlotTurbo™ Mini PVDF Transfer Packs (BIORAD). The unoccupied membrane sites were blocked with T-TBS 1X (Tween 0.1%) and 5% non-fat dry milk to prevent nonspecific binding of antibodies and probed with specific primary antibodies overnight at 4 °C. This was followed by incubation with the

respective secondary antibodies. The antibody-antigen complexes were detected by chemiluminescence with Clarity™ Western ECL Substrate (BIO-RAD).

3D cell Cultures: Method. A rotary cell culture system (RCCS) (Synthecon) endowed with gas-permeable culture vessels and placed inside a humidified 37°C, 1% CO2 incubator, has been used. The monolayer medium was used also for cell growth in the RCCS. A549 single cell suspensions were placed in the rotating chamber at an initial speed of 10 rpm. The culture medium was changed every 4 days and tumour spheroids with a diameter ranging from about 500 µm to 1.2 mm will be obtained in 15–20 days.

3D Cell Cultures: Clarification protocol. To render A549 spheroids transparent we set up and optimezed a clarification protocol for use with X-ClarityTM Tissue Clearing System (Logos Biosystems). Fixed A549 spheroids/organoids were incubated for 22 hours at 4°C in the X-CLARITY Hydrogel-Initiator solution according to manufacturer's instructions. Subsequently, samples were first polymerized with the X-ClarityTM Polymerization System and then immersed in an Electrophoretic Tissue Clearing Solution (Logos Biosystems) and placed for 8 hours in the X-ClarityTM Tissue Clearing System, which is able to actively extract lipids through electrophoresis to create a stable and optically transparent tissue-hydrogel hybrid that is chemically accessible for multiple rounds of antibody labelling and imaging. At the end of the procedure A549 spheroids/organoids, washed with PBS to remove clearing solution, can be stored at 4°C until use.

3D Cell Cultures: High Resolution Imaging on 3D cell cultures. Confocal analysis was performed using a FluoView FV1000 confocal microscope (Olympus) equipped with silicone immersion objectives (30x and 60X) that provide long working distances (0.8 mm and 0.3 mm, respectively. Moreover, the refractive index of silicone oil (n = 1.40) is closer to the refractive index of X-CLARITY mounting solution (n = 1.46), reducing the spherical aberration caused by mismatched refractive indices. Confocal images were processed using Richardson-Lucy deconvolution algorithm.

EVs characterization: Isolation of EVs. Briefly, cell lines were grown to 70% confluence, at which point the growth medium was replaced with FBS-depleted equivalent medium and exposed to radiation treatment. After 72h, 200 ml of conditioned supernatant were collected and centrifuged at 500 g for 5 min at 4°C to pellet cellular contaminant, followed by 3000 g for 30 min at 4°C to pellet cell debris. The supernatants were centrifuged at 18,000 g for 30 min and subsequently for 30 min at 10,000 g. The resulting supernatants was ultra-centrifuged two times at 100,000 g for 70 min and 120 min respectively at 4°C. The final EVs pellet was solubilized in 100 µl of filter-sterilized PBS. EVs were used immediately or stored at -80°C.

EVs Characterization: Nanoparticle Tracking Analysis (NTA). Nanoparticle tracking analysis was applied to determine particle size and concentration of all samples. Purified EVs were diluted in 0.22 µm filtered PBS to an appropriate concentration before analysis, loaded into the sample chamber of a NanoSight LM10 instrument (Malvern, UK). At least three videos of either 30 or 60 seconds were recorded of each sample. Temperature was kept constant throughout the measurements. Data analysis was performed with NTA 3.1 software. Data is presented as the average and standard deviation of the three video recordings. When samples contained large numbers of particles, they were diluted before analysis and the relative concentration was then calculated according to the dilution factor. Control 100 and 400 nm beads were supplied by Malwern (UK).

EVs Characterization: Surface Markers (by western blot analysis).

EV lysates were prepared in Lysis buffer according the Peña-Llopis protocol (Peña-Llopis S & Brugarolas J. Nat Protoc 2013) and quantified using PierceTM BCA Protein Assay (Thermo Scientific). Next, the lysates were subjected to SDS-PAGE and transferred to PVDF membranes (BIO-RAD). Membranes were then incubated with the primary antibodies overnight at 4°C. The following primary antibodies were used: CD9 (RRID:AB_2798139; 1:1000 dilution), Annexin V (RRID:AB_10950499; 1:1000 dilution) and CD63 (RRID:AB_648179; 1:200 dilution). After incubation with the specific HRP-conjugated antibody (RRID:AB_631746; 1:5000 dilution; RRID:AB_631736; 1:5000 dilution), the chemiluminescent signal was detected using SuperSignal™ West Femto Maximum Sensitivity Substrate (Thermo Scientific).

Macrophage polarization by EVs. Human monocytic THP-1 cells were differentiated into macrophages by 24 h incubation with 150 nM phorbol 12-myristate 13-acetate followed by 24 h incubation in RPMI medium. Macrophages were polarized in M1 macrophages by incubation with 20 ng/ml of IFN-γ and 10 pg/ml of LPS. Macrophage M2 polarization was obtained by incubation with 20 ng/ml of interleukin 4 and 20 ng/ml of interleukin 13. Finally, macrophages were exposed to EVs isolated from serum-free medium of irradiated A549 cells. Analysis of gene expression levels of M2 polarization markers PPAR-alpha, TNF-alpha, IL-1β, STAT-1 was performed on THP-1 cells exposed to each condition above described (see Supplementary Table 1 for specific primers).

Evaluation of Inhibitory effects of EVs. 500 cells of non-irradiated A549 were seeded in 10-cm^2 dishes in 500 ml of medium and exposed to EVs isolated from irradiated A549 cells for 14 days. Eventually, the resulting colonies were fixed and stained using 0.5% crystal violet in 25% methanol; colonies with more than 50 cells were quantified by two independent observers under

inverted microscope (Olympus IX51 microscope, Olympus Corporation, Tokyo, Japan). Five series of samples were prepared for each treatment dose.

DATA IMAGING ANALYSIS

Foci analysis procedure. Cells were imaged by fluorescent confocal microscopy in at least 15 optical section, eventually summarised in 2-D single-channel 12-bit maximum projection images (MIPs), further quantized to 8-bit for de-noising purposes. RGB fusion of 8-bit MIPs and conservative thresholding of the resulting image allow for the safest possible cell borders detection. The MIP of the blue channel, informative of DAPI staining, is conservatively thresholded by maximum entropy method to outline the nuclear membrane. Single-cell nuclei and cytoplasms are then segmented by in-house adaptive algorithms, which in order allow for: a) automated removal of noise arising from fixation and staining procedures; b) automated detection and exclusion of partly imaged cells; c) separation of touching cells. Estimating cell borders from indirect staining yields cells detected as fragmented objects, which are later recomposed on the basis of their geodesic distance. Sub regional cell foci segmentation is finally carried out in three steps, where: a) a top-hat transform image pre-processing is performed basing on MIP content and acquisition parameters; b) single cell nucleus and cytoplasm masks previously generated are applied on original MIPs to extract radiometric information; c) thresholds for regional foci segmentation are found by adaptive application of triangle and ISODATA thresholding. Generated foci masks are applied to the original 12-bit MIPs to extract the intensity values of nuclear and cytoplasmic single foci at cell level. The medians of foci intensity distributions were tested with a) one-sample Wilcoxon signed-rank test; b) unpaired two-sample Wilcoxon-Mann-Whitney rank test; c) unbalanced two-way ANOVA. For all the tests p-value $\leq .01$ was considered for statistical significance.

Sample size of tested cell populations are reported in the following tables:

Table S2. Sample size of populations for CD63 fluorescence quantitation.

	UnIR	10 Gy	20 Gy
A549	41	46	38
H1299	92	75	44
A549 sh/p53	50	33	58

Table S3. Sample size of populations for RNA:DNA hybrids fluorescence quantitation.

	UnIR	UnIR Exo	10 Gy	20 Gy
A549	17		8	10
H1299	64		50	67
A549sh/p53	14		10	11
A549 Exo	23	27	35	42

Table S4. Sample size of populations for ORF1 fluorescence quantitation.

	UnIR	10 Gy	20 Gy
A549	44	44	45
A549 sh/p53	57	74	49

Statistical Analysis of Nuclear foci Intensity Figure 7A

Raw data (GrandMedian intensity ± MAD):

Table S5. A549

MEDIAN	MAD	MEAN	STD	VARIANCE
846,0	78,0	862,5	183,6	33704,0
903,0	170,0	866,7	231,6	53632,1
1087,0	98,0	1084,3	160,1	25642,8

Table S6. A549sh/p53

MEDIAN	MAD	MEAN	STD	VARIANCE
1349,0	350,0	1270,2	631,0	398134,6
1162,0	302,0	1230,1	439,4	193053,4
1281,5	510,0	1236,1	576,5	332373,1

Table S7. p-values (two-sample Wilcoxon-Mann-Whitney rank sum tests, α=0.05):

	UnIR – 10 Gy	UnIR – 20 Gy	10 Gy – 20 Gy
**A549**	0,92873	$2,6*10^{-4}$	$7,6*10^{-4}$
**A549sh/p53**	0,72139	0,99000	0,97586

Co-localization analysis

A549 WT and A549 siP53 cells were plated at 3x104/well on a glass slide and treated with radiation. After 72h from radiation treatment cells were fixed in paraformaldehyde 4% for 15 minutes at room temperature, washed three times in PBS 1X and blocked for 1 hour in blocking solution (1X PBS/5% normal goat serum/0.3% Triton X-100) and then incubated overnight at 4°C with primary rabbit anti-ORF1 antibody (clone JH73, kindly provided by Prof. Han Jeffrey, Department of Biochemistry and Molecular Biology Tulane University School of Medicine, Tulane Cancer Center, New Orleans, USA) at dilution 1:200 and mouse anti-S9.6 (Kerafast) at dilution 1:100. After washing in PBS 1X, slides were incubated 1 hour at room temperature with secondary goat anti-rabbit Alexa Fluor™ 647 (Invitrogen™) at dilution 1:300 and with secondary goat anti-mouse Alexa Fluor™ 546 (Invitrogen™) at dilution 1:250. Slides were then washed 5 times with PBS 1X and then stained with DAPI and mounted with ProLong™ Diamond Antifade Mountant (Invitrogen™, Thermo Fisher Scientific, cat n. P36961). The confocal imaging was performed with a Nikon A1 confocal laser scanning microscope, equipped with a 60X oil objective (1.4 NA), using 405, 561 and 640 nm laser lines. The colocalization of the fluorochromes was evaluated by comparing the equivalent pixel positions of red and green signals in each of the acquired images (optical sections). A two-dimensional scatter plot diagram of the individual pixels from the paired images was generated and a threshold levels of signal to be included in the analysis was selected. Pixels with intensity values greater than 5% grey levels (on a scale from 0 to 4096) were selected for both signals, and colocalization binary maps that indicate regions containing highly colocalized signals were generated (Riccio et al. 2004 Humana Press). Moreover, the colocalization was quantified using Mander's Overlap coefficient and expressed as percentage ± SD. Image analysis (volume measurements and 3D object count) was performed using NIS-Elements Advanced Research software (Nikon).
